# Phosphatidylserine Increases IKBKAP Levels in Familial Dysautonomia Cells

**DOI:** 10.1371/journal.pone.0015884

**Published:** 2010-12-29

**Authors:** Hadas Keren, Maya Donyo, David Zeevi, Channa Maayan, Tal Pupko, Gil Ast

**Affiliations:** 1 Department of Human Molecular Genetics and Biochemistry, Sackler Faculty of Medicine, Tel Aviv University, Ramat Aviv, Israel; 2 Department of Cell Research and Immunology, George S. Wise Faculty of Life Sciences, Tel Aviv University, Ramat Aviv, Israel; 3 Department of Pediatrics, Hadassah University Hospital, Mount Scopus, Jerusalem, Israel; Centre de Regulació Genòmica, Spain

## Abstract

Familial Dysautonomia (FD) is an autosomal recessive congenital neuropathy that results from abnormal development and progressive degeneration of the sensory and autonomic nervous system. The mutation observed in almost all FD patients is a point mutation at position 6 of intron 20 of the *IKBKAP* gene; this gene encodes the IκB kinase complex-associated protein (IKAP). The mutation results in a tissue-specific splicing defect: Exon 20 is skipped, leading to reduced IKAP protein expression. Here we show that phosphatidylserine (PS), an FDA-approved food supplement, increased IKAP mRNA levels in cells derived from FD patients. Long-term treatment with PS led to a significant increase in IKAP protein levels in these cells. A conjugate of PS and an omega-3 fatty acid also increased IKAP mRNA levels. Furthermore, PS treatment released FD cells from cell cycle arrest and up-regulated a significant number of genes involved in cell cycle regulation. Our results suggest that PS has potential for use as a therapeutic agent for FD. Understanding its mechanism of action may reveal the mechanism underlying the FD disease.

## Introduction

Familial dysautonomia (FD) is an autosomal recessive congenital neuropathy that occurs almost exclusively in the Ashkenazi Jewish population with a carrier frequency between 1 in 27 to 1 in 32 [1], [2]. Ashkenazi Jews of Polish descent have a higher carrier frequency of 1 in 18 [3]. FD results from abnormal development and progressive degeneration of the sensory and autonomic nervous system. Patients are severely affected with a variety of symptoms in most body systems. Among these symptoms are gastrointestinal and cardiovascular dysfunction, vomiting crises, abnormal sensitivity to pain and temperature, and recurrent pneumonia. Despite recent advances in patient management, about 50% of patients die before the age of 40 [4], [5], [6].

**Figure 1:**
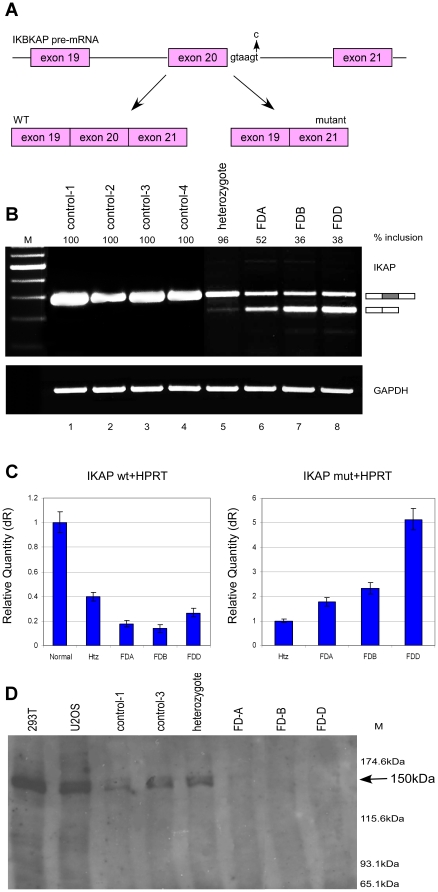
Expression of IKAP mRNA and protein in FD cells. (**A**) Schematic diagram illustrating the area in *IKAP* responsible for FD. The FD mutation at position six of exon 20 splice donor site is shown by an arrow. In FD patients, two mRNA isoforms, one containing exon 20 and one without, can be present. (**B**) RT-PCR analysis of *IKAP* mRNA. RNA was extracted from control, heterozygous and FD cells and the endogenous splicing products were separated on a 2% agarose gel after RT-PCR reaction using primers to exon 19 and 21. Isoforms were quantified using ImageJ. GAPDH was used as control for cDNA amounts. (**C**) QPCR analysis of the *IKAP* mRNA. Left side: Level of exon 20 inclusion isoform (wt). Relative quantity represents normalization to control cells. Right side: Level of exon 20 skipped isoform (mut). Relative quantity represents normalization to heterozygous (Htz) cells. All values were normalized to *HPRT* mRNA. QPCR experiments were amplified in triplicate; results shown are mean values ± SD. (**D**) Analysis of IKAP protein levels. Western blotting of extracts from the indicated cell lines using an anti-IKAP antibody (Santa Cruz Biotechnology, D-17). Band intensities were quantified using ImageJ.

The gene associated with the disease was linked to chromosome 9q31 and identified as the *IKBKAP* gene. This gene encodes the IκB kinase complex-associated protein (IKAP; for simplicity, *IKAP* is used rather than *IKBKAP* to refer to the mRNA encoded by this gene). The point mutation observed in almost all FD patients (>99.5%) is a change from T to C at position 6 of the 5′ splice site (5′ss) of intron 20 [7], [8]. The mutation results in a shift from constitutive inclusion to alternative splicing of exon 20 (Figure 1A). The splicing defect in FD is tissue specific. Tissues from the brain and nervous system express primarily mutant *IKAP* mRNA (skipping of exon 20), while other tissues express both wild-type and mutant mRNA in different ratios [4], [9], [10]. The skipped isoform has a frameshift relative to the wild-type mRNA that results in a premature stop codon, leading to considerably reduced IKAP expression [9], [10]. The mutant transcript is a potential target for degradation by the nonsense mediated decay (NMD) pathway [11]. In our system, treatment with cycloheximide, an inhibitor of NMD, did not alter the level of the mutant transcript (data not shown); however, this is not consistent with observations from another study [12].

The IKAP protein is a 1332 amino acid, 150-kDa protein that is highly conserved in eukaryotes [13], [14]. The function of IKAP has been a subject of much research but is still obscure. Based on homology to a yeast protein, ELP1, and co-purification with human Elongator [13], IKAP is thought to be a subunit of the Elongator complex, which assists RNA polymerase II in elongation of transcription in the nucleus [13], [15], [16]. There is evidence that in the cytosol IKAP is involved in regulation of the c-Jun N-terminal kinase (JNK) signaling pathway [14], tRNA modification [17], exocytosis [18], cell adhesion, migration of cells and reorganization of actin in the cytoskeleton [19], [20]. IKAP may also play a role in oligodendrocyte differentiation and/or myelin formation [21] and in p53 activation [22]. IKAP is also crucial for vascular and neural development during embryogenesis [23].

Based on our current knowledge of FD and what is known so far about IKAP, we presume that the key for effective therapy of FD is increasing the amount of the normal, functional IKAP protein. We found that an FDA-approved food supplement, phosphatidylserine (PS), increased the amount of wild-type *IKAP* mRNA in FD cell lines. Further, long-term treatment of FD cells led to a significant increase in the amount of IKAP protein. Untreated FD cells accumulated at the G1 state with lower levels of cells in S and G2 states. PS treatment released this blockage, and this was associated with elevation in expression of genes involved in cell cycle regulation. Overall, our data indicate that PS has promise for treatment of FD patients.

## Results

To examine the effect of potential drugs on the splicing of the *IKAP* mRNA we used three FD cell lines derived from three FD patients (termed FDA, FDB and FDD). In addition, a cell line derived from a parent of an FD patient, and therefore heterozygous for the FD mutation, and four matched cell lines derived from healthy individuals were used as controls. Analysis of the splicing pattern of exon 20 was performed by RT-PCR using primers to the endogenous *IKAP* mRNA. Figure 1B reveals that exon 20 was constitutively spliced in the control cell lines (lanes 1–4); 96% inclusion was observed in the heterozygous cells (lane 5), and from 38% to 52% inclusion was observed in the three FD cell lines (lanes 6–8). Figure 1B also demonstrates that *IKAP* mRNA was either expressed at higher levels or was considerably more stable in the control compared to the FD-derived cell lines. The levels of the wild-type and mutant isoforms were further quantified by real-time quantitative PCR (QPCR) analysis (Figure 1C). FD cells expressed considerably lower amounts of wild-type *IKAP* mRNA compared to control cells (Figure 1C, left side). The heterozygous cell line expressed 2.5-fold less wild-type *IKAP* mRNA than did control cells. FD cells, FDA, FDB and FDD, expressed 5.7-, 7.2- and 3.8-fold less, respectively, wild-type *IKAP* mRNA than did control cells. As shown in Figure 1C, right side, the skipped *IKAP* isoform was expressed at different levels in the FD cell lines compared to the heterozygous cell line. No skipped isoform was detected in control cells (data not shown).

In order to characterize the IKAP protein levels, a western blot analysis was conducted (Figure 1D). The control cell lines and the heterozygous cell line expressed the wild-type IKAP protein at the expected size of 150 kDa at similar levels (1.2 less IKAP protein in heterozygous cells). However, despite the presence of both wild-type and mutant mRNA isoforms as shown by RT-PCR, in FD cells only a very faint 150-kDa band was observed. Between 4 and 5 fold less IKAP protein was observed in FD cells than in control cells. No product of the size expected for the truncated protein, 79 kDa, was detected, in agreement with previous reports [10], [24].

**Figure 2:**
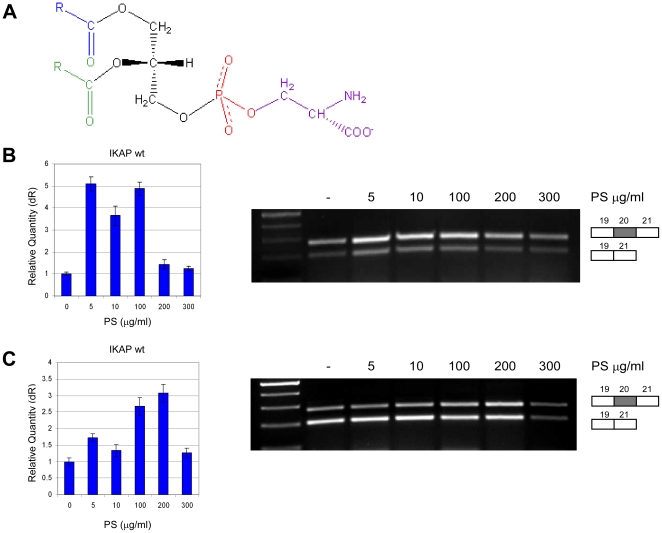
PS raises *IKAP* mRNA levels in FD cell lines. (**A**) Chemical structure of PS. (**B**) FDA cells or (**C**) FDB cells were treated with 0, 5, 10, 100, 200 and 300 µg/ml PS. RNA was extracted after 24 hr for FDA cell line and after 48 hr for FDB cell line. Left side: QPCR analysis of the level of exon 20 inclusion isoform (wt). Data were normalized to levels in untreated control cells. Right side: RT-PCR analysis of the splicing of the endogenous *IKAP* mRNA in FD cells. All splicing products were separated on a 2% agarose gel after RT-PCR reaction using primers to exons 19 and 21. The PCR products were eluted and sequenced. All experiments were repeated independently three times, and the results shown are representative of an average experiment. QPCR experiments were amplified in triplicate; results shown are mean values ± SD.

FD is characterized by dysfunction of the autonomic and sensory nervous system resulting from incomplete neuronal development and progressive neuronal degeneration [1]. We therefore sought substances that would affect neuronal function and would be safe for immediate testing in FD patients. We first examined two food supplements, one based on choline and one based on serine. The choline-based substance had little effect on the level of *IKAP* mRNA. However, the serine-based substance, phosphatidylserine (produced by Enzymotec under the Sharp•PS® brand), significantly increased *IKAP* mRNA and IKAP protein levels in cells derived from FD patients. PS, a nutritional supplement, is a major component of every living cell, especially neuronal cells [25], and slows cognitive degeneration in human subjects [26], [27], [28]. We found that in FD cell lines PS significantly raises *IKAP* mRNA and protein levels. PS, shown in Figure 2A, is used worldwide and is considered by the FDA as a safe and lawful dietary supplement. PS was added to FD cells at concentrations ranging from 0–300 µg/ml. The cells were harvested and RNA was extracted 24 and 48 hr following the addition of PS for each cell line; *IKAP* mRNA was analyzed by RT-PCR analysis and QPCR. The highest effect of PS on *IKAP* mRNA levels was obtained at different time points for each cell line. After 24 hr of treatment the best results were obtained for cell line FDA; we observed about a 5-fold increase in the amount of wild-type *IKAP* mRNA after treatment with 5, 10 or 100 µg/ml PS compared to levels in FDA cells treated with the solvent only (Figure 2B). At 200 µg/ml PS, the increase was 1.5 fold, and at 300 µg/ml a 1.2-fold increase in wild-type *IKAP* mRNA levels was observed. A possible explanation for lower efficacy at higher PS concentration is the toxicity of the solvent at high volumes, although the solvent itself did not affect *IKAP* mRNA levels (data not shown). After 48 hr of treatment, best results were obtained for cell line FDB; *IKAP* mRNA levels peaked at about 3-fold higher than levels in untreated cells at 100 and 200 µg/ml PS (Figure 2C). PS did not affect the ratio of the included to skipped mRNA isoforms but rather elevated the total amount of both isoforms. In FDD cells, PS had variable effects on *IKAP* mRNA (Figure S1). In the heterozygous cell line, PS treatment induced a slight increase in *IKAP* mRNA (data not shown). These results indicate that the effect of PS on the level of *IKAP* mRNA differed among FD cell lines.

**Figure 3:**
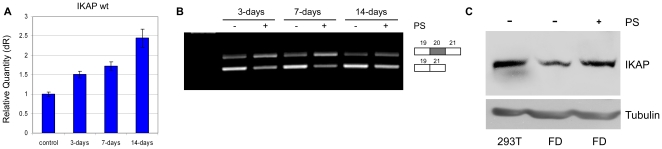
PS raises IKAP mRNA and protein levels following long-term treatment. PS was added to FDB cells at a concentration of 100 µg/ml. Every two days the medium was replaced and fresh PS was added. RNA was extracted 3, 7 and 14 days following the initial addition of PS. All experiments were repeated independently three times, and the results shown are representative of an average experiment. (**A**) QPCR analysis of the level of exon 20 inclusion isoform (wt). Data were normalized to levels in untreated control cells harvested on the same day. QPCR experiments were amplified in triplicate; results shown are mean values ± SD. (**B**) RT-PCR analysis of the splicing of the endogenous *IKAP* in FDB cells. All splicing products were separated on a 2% agarose gel after RT-PCR reaction using primers to exons 19 and 21. The PCR products were eluted and sequenced. (**C**) Western blotting using an anti-IKAP antibody (BD-Bioscience) of FDB cells treated with PS for two weeks. The exposure of the analysis of 293T cells was reduced to one fourth of its original amount in order to avoid intense background and to better visualize the effect of PS. Band intensities were quantified using ImageJ.

In clinical studies, PS efficacy increased as treatment was prolonged [26]. We thus examined the effect of PS on the level of *IKAP* mRNA in FDB cells after 3, 7 and 14 days of treatment. The level of *IKAP* mRNA was evaluated by QPCR (Figure 3A) and RT-PCR (Figure 3B). As shown in Figure 3A, PS treatment increased the level of *IKAP* mRNA by 1.5 fold at 3 days, 1.7 fold at 7 days, and 2.5 fold after 14 days compared to levels in untreated cells. Proteins were harvested two weeks after the addition of the supplement and IKAP levels were analyzed by western blot. The addition of PS increased the amount of IKAP protein by 2.3 fold in FD cells treated with PS relative to untreated FD cells (Figure 3C). It should be noted that the antibody used in this western blot (from BD Bioscience) can detect only the wild-type IKAP protein. The antibody used in Figure 1D to detect the level of IKAP protein in the different cell lines (from Santa Cruz Biotechnolgy) can theoretically detect both the wild-type and truncated IKAP protein.

**Figure 4:**
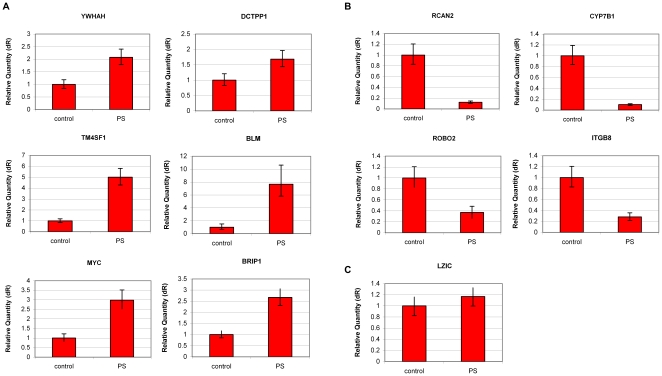
Validation of gene expression microarray analysis by QPCR. FDB cells were treated with 100 µg/ml PS. RNA was extracted 24 hr following the addition of the supplement. A portion of each RNA sample was used for microarray analysis, and an aliquot was saved for experimental validation. QPCR analysis of genes shown by the gene expression microarray analysis to be (**A**) up-regulated or (**B**) down-regulated as a result of PS treatment. Each panel shows the levels in FDB cells after PS treatment relative to untreated FDB cells. (**C**) All values were normalized to a control, the *LZIC* gene transcript, that did not change as a result of PS treatment. QPCR experiments were amplified in triplicate; results shown are mean values ±S.D.

In order to test whether expression of genes in addition to IKAP was altered as a result of PS treatment, we performed a human gene expression microarray analysis (Human Gene 1.0, Affymetrix) of cDNA samples from FDB cells treated with 100 µg/ml PS. Using SAM (Significance Analysis of Microarrays) analysis, we identified 877 genes with significantly different levels of expression following PS treatment: 441 genes were up-regulated and 436 genes were down-regulated. These genes are listed in Figure S2 of the Supplementary Material. We confirmed the effect of PS on six significantly up-regulated genes (*YWHAH*, *TM4SF1*, *MYC*, *DCTPP1*, *BLM* and *BRIP1*) and four significantly down-regulated genes (*RCAN2*, *ROBO2*, *CYP7B1*, and *ITGB8*) using QPCR (Figure 4). All values were normalized to levels of *LZIC*, which were unchanged by PS treatment. A gene ontology (GO) enrichment analysis of these genes was performed using the Database for Annotation, Visualization, and Integrated Discovery (DAVID) [29]. A significant GO enrichment was observed for the up-regulated genes coding for proteins involved in regulation of the cell cycle and DNA metabolic processes (Table 1). Complete tables are presented in Figure S3. As a group, the down-regulated genes did not present highly significant GO enrichment; most of them function in developmental processes (data not shown). Using the Kyoto Encyclopedia of Genes and Genomes (KEGG) pathways database in DAVID we also observed a significant enrichment for genes involved in the signaling pathways for pyrmidine and purine metabolism, as well as for genes known to be involved in base excision repair (Table 2).

**Table 1:** GO analysis for up-regulated genes.

Cluster 1	Enrichment score−33.994	
GO term	p-value^1^	# genes^2^
Cell cycle	7.15E-41	96
Cell cycle phase	6.16E-36	69
Cell cycle process	2.08E-34	77
M phase	6.40E-34	61
Mitotic cell cycle	6.44E-30	60
Mitosis	4.02E-29	48
Nuclear division	4.02E-29	48
M phase of mitotic cell cycle	9.58E-29	48
Organelle fission	2.76E-28	48
Cell division	3.79E-24	49

Cluster 2	Enrichment score−25.16	
GO term	p-value^1^	# genes^2^
DNA metabolic process	5.93E-35	74
Response to DNA damage stimulus	2.65E-21	51
DNA repair	5.25E-21	45
Cellular response to stress	1.89E-13	51

Cluster 3	Enrichment score−22.12	
GO term	p-value^1^	# genes^2^
Nuclear lumen	3.65E-27	104
Intracellular organelle lumen	2.43E-22	108
Membrane-enclosed lumen	4.98E-22	110
Organelle lumen	1.60E-21	108
Nucleoplasm	1.44E-13	62
Nucleolus	1.05E-11	52

Cluster 4	Enrichment score−21.08	
GO term	p-value^1^	# genes^2^
Chromosome	2.63E-31	65
Chromosomal part	7.37E-29	58
Chromosome, centromeric region	5.19E-18	29
Condensed chromosome	2.93E-15	27
Condensed chromosome, centromeric region	4.83E-15	21
Condensed chromosome kinetochore	6.45E-15	20
Condensed chromosome kinetochore	1.44E-13	21
Chromosome segregation	2.21E-12	21

Enriched categories were identified using DAVID to cluster differentially up- and down-regulated genes into functional categories using GO identification terms. Significant GO enrichment (p-value <0.05 after FDR multiple testing correction) was observed only for the up-regulated genes.

1 FDR multiple testing correction.

2 Number of identified genes in Gene Ontology (GO) category.

Enriched categories were identified using DAVID; See supplementary file 3 for complete tables.

**Table 2:** Signaling pathways enrichment of genes up-regulated by PS treatment.

Cluster 1	Enrichment score−4.42	
Term	p-value^1^	# genes^2^
Pyrimidine metabolism	0.001268	13
Purine metabolism	0.03833	14

Cluster 2	Enrichment score−4.05	
Term	p-value^1^	# genes^2^
Base excision repair	0.011218	8

Enriched signaling pathways were identified using the KEGG pathways database in DAVID to cluster up- and down-regulated genes. Significant KEGG enrichment (p-value <0.05 after FDR multiple testing correction) was observed only for the up-regulated genes.

1 FDR multiple testing correction.

2 Number of identified genes in the KEGG pathway category.

Enriched categories were identified using DAVID.

**Figure 5:**
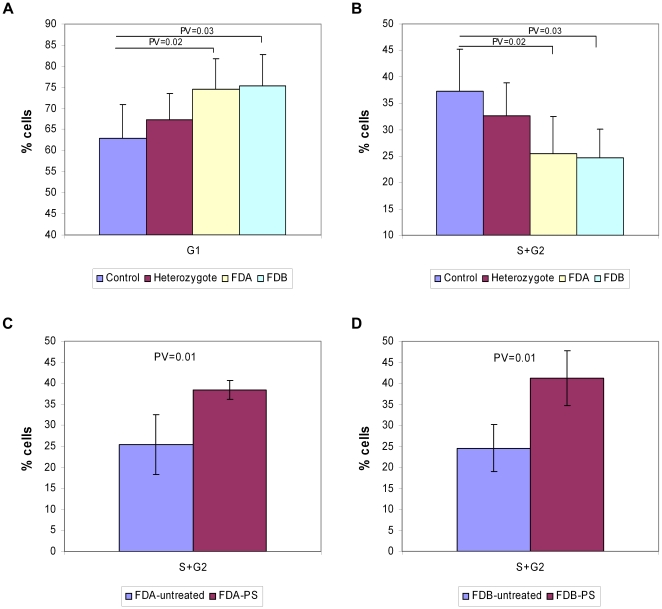
PS alters the cell cycle distribution of FD cells. One day prior to treatment 750,000 cells were seeded. The cells were treated with 100 µg/ml PS for 24 hr and then fixed and stained with PI according to the flow cytometry protocol. The percentage of cells (**A**) at the G1 stage and (**B**) at the growth stages, S+G2, of untreated control, heterozygous, FDA and FDB cells. The percentages of cells at the growth stages, S+G2, of (**C**) FDA or (**D**) FDB cells following PS treatment compared to untreated FD cells. All experiments were repeated independently four times, and the results shown are mean values ± SD. P-values of significant results are indicated.

Due to the fact that a significant number of genes up-regulated by PS treatment are involved in cell cycle regulation, we tested the effect of PS on the cell cycle distribution of FD cells using propidium iodide (PI) staining and flow cytometry. Cell cycle analysis of untreated FD cells revealed that a significantly higher fraction of the cells were in the G1 stage compared to control or heterozygous cells (Figure 5A). A lower fraction of the FD cells were in the S+G2 stages, 1.5-fold less than in the control cells, indicating that a low number of FD cells are in the dividing state (Figure 5B). Treatment of FD cells with PS significantly raised the fraction of cells in S+G2 stages by 1.5 fold compared to untreated FDA cells (Figure 5C) and 1.7 fold compared to untreated FDB cells (Figure 5D). These results indicate that PS releases FD cells from cell cycle arrest.

Since PS treatment increased the amount of the wild-type *IKAP* mRNA and protein present in FD cells, we obtained three additional food supplements and tested their effect on splicing. The first is produced under the name Sharp·PS GOLD4508P and is a proprietary conjugate of PS and docosahexaenoic acid (DHA), an omega-3 fatty acid. Sharp·PS GOLD resembles the functional form of natural (brain) PS, increases DHA availability in the brain, and acts to increase cognitive abilities [30]. The second substance tested was L-α-glycerophosphorylcholine (GPC), a dietary supplement reported to improve mental performance that is marketed as SharpGPC 85F. GPC serves as a precursor for reconstituting a nerve cell membrane component. Clinical trials over the past two decades have demonstrated that treatment of subjects with adult-onset dementia disorders with GPC at 1,000–1,200 mg per day protects against cognitive impairment characteristics of dementia disorders [31]. Third, we tested Krill oil+4225F, a proprietary complex of marine-derived DHA and eicosapentanoic acid (EPA), delivered as triglycerides or attached to phospholipids. Krill oil+4224F also contains a significant amount of astaxanthin. A similar combination improves blood-lipid markers, including LDL, HDL and triglycerides, and in clinical studies, Krill oil has greater potency than omega-3, due to its unique structure and composition [32], [33].

**Figure 6:**
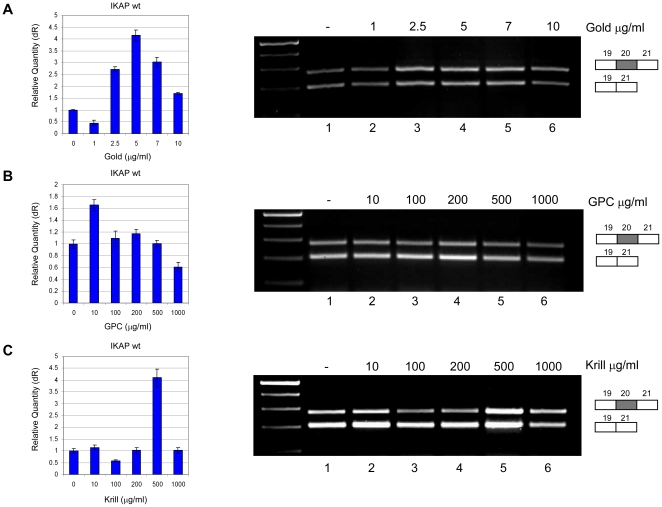
Other supplements also increase *IKAP* mRNA levels in an FD cell line. (**A**) Sharp·PS GOLD4508P (Gold), (**B**) SharpGPC 85F (GPC), or (**C**) Krill oil+4225F (Krill oil) were added to FDB cells at the indicated concentrations. RNA was extracted 24 hr following the addition of the supplement. Left side: QPCR analysis of the level of exon 20 inclusion isoform (wt). Data were normalized to that of untreated control cells. Right side: RT-PCR analysis of the splicing of *IKAP* in FDB cells. All splicing products were separated on a 2% agarose gel after RT-PCR reaction using primers to exons 19 and 21. The PCR products were eluted and sequenced. All experiments were repeated independently three times, and the results shown are representative of an average experiment. QPCR experiments were amplified in triplicate; results shown are mean values ± SD.

FD cell lines were treated with these three formulations, and the effects on the splicing of *IKAP* mRNA were analyzed by RT-PCR and QPCR (Figure 6). At 5 µg/ml, the Sharp·PS GOLD supplement increased the level of *IKAP* mRNA level 4.2 fold compared to levels in untreated cells (Figure 6A). Sharp·PS GOLD increased the level of the wild-type *IKAP* mRNA at one twentieth the effective concentration of PS. Treatment with GPC did not significantly increase the amount of wild-type *IKAP* mRNA (Figure 6B). At 500 µg/ml, Krill oil increased levels of the wild-type *IKAP* mRNA significantly (Figure 6C).

**Figure 7:**
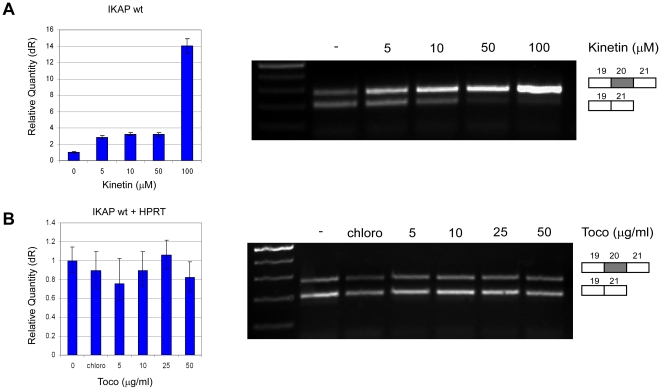
The effect of substances already tested in FD models or patients on *IKAP* mRNA levels. (**A**) Kinetin or (**B**) tocotrienol (Toco) were added to FDB cells at the indicated concentrations. RNA was extracted 24 hr following the addition of the substance. Left side: QPCR analysis of the level of exon 20 inclusion isoform (wt). Data were normalized to that of untreated control cells. Right side: RT-PCR analysis of the splicing of the endogenous *IKAP*. All splicing products were separated on a 2% agarose gel after RT-PCR reaction using primers to exons 19 and 21. The PCR products were eluted and sequenced. All experiments were repeated independently three times, and the results shown are representative of an average experiment. QPCR experiments were amplified in triplicate; results shown are mean values ± SD.

To evaluate PS with respect to other substances already tested for FD treatment, we treated the FD cells with kinetin and tocotrienol. Kinetin is a plant cytokinin, which was reported to rescue mRNA splicing of *IKAP ex vivo* [12], [24], [34] and in patients heterozygous for the FD mutation [35]. A significant increase in wild-type *IKAP* mRNA levels and a shift towards a higher level of exon 20 inclusion was observed with an optimal concentration of 100 µM of kinetin (Figure 7A), with no toxicity observed. In contrast, tocotrienol, a member of the vitamin E family that was reported to induce IKAP expression in FD cells [36], [37], [38], did not affect *IKAP* mRNA expression in our system (Figure 7B); this observation is similar to that of another publication [39]. These results indicate that the FD cells used in this study are sensitive to kinetin treatment but not to tocotrienol.

## Discussion

FD is caused by a mutation in the *IKBKAP* gene that leads to aberrant splicing. The skipped isoform produced from the mutant gene is unable to produce normal IKAP protein, and the levels of normal *IKAP* mRNA in FD patients fail to provide sufficient amounts of IKAP protein. Subjects that are heterozygous for the FD mutation express a lower level of wild-type *IKAP* mRNA than do normal subjects (Figure 1B, 1C) but do not display any of the clinical symptoms of FD [35]. We sought substances already proven safe for clinical use that would increase the levels of wild-type *IKAP* mRNA in cell lines created from cells taken from FD patients in order to identify potential treatments.

We demonstrated that phosphatidylserine significantly increased the levels of normal *IKAP* mRNA and protein. The FDA has allowed two qualified health claims for the use of PS: PS may improve the risk of cognitive dysfunction and may reduce the risk of dementia in the elderly [27]. Use of PS derived from bovine brain cortex has been shown to slow cognitive degeneration and in some cases improve cognitive function, especially in adults suffering from mild dementia [26], [27], [28]. The substance is also effective in treating attention deficit hyperactivity disorder (ADHD) in children [40]. Plant-derived PS was shown to be effective for treatment of age-related cognitive decline in an open trial study [41]. PS derived from soy lecithin is currently given orally to adults at a dosage of 200–300 mg per day and to children at 520 mg per day. The substance has been tested in humans and animals at a higher dose than recommended for use in humans with no toxicity observed [42], [43], [44], [45]. The PS/DHA conjugate (Sharp·PS GOLD) increased the level of *IKAP* mRNA at much lower concentrations than the effective concentrations of PS in the FD cells used in our study. This is probably due to the combined, and possibly synergistic, effects of PS and DHA. This implies that analogs of PS should be further investigated.

The effect of PS on IKAP levels was not related to changes in the ratio between exon 20 inclusion and skipping. Rather PS acted by increasing levels of transcription of this gene; increased levels of mRNA led to elevation of the level of the normal IKAP protein. In an attempt to elucidate the mechanism of action of PS, we compared gene expression in FD cells in the presence and absence of PS. A significant number of the genes up-regulated following PS treatment are involved in cell cycle regulation. That FD cells have improper regulation of the cell cycle was confirmed by examining the cell cycle distribution of FD cells. Compared to control or heterozygous cells, a lower percentage of FD cells were in growth phases. A significant fraction of FD cells were in G1 compared to control cells; this suggests that exit from G1 into the S phase is impaired in FD cells. This may mean that the IKAP protein is directly or indirectly involved in cell cycle checkpoint regulation, and the reduced levels of IKAP in FD cells results in abnormal growth of cells. PS treatment of FD cells elevated wild-type *IKAP* mRNA and protein levels and resulted in a cell cycle distribution similar to that of the control cells. A significant number of genes up-regulated following PS treatment are also involved in DNA metabolic processes and in DNA repair mechanisms. Upon increases in cellular metabolism and synthesis of nucleotides, transcription by RNA polymerase II increases. This enhancement of RNA polymerase II activity results in a meaningful increase in levels of mRNAs from genes that are usually under expressed, such as *IKAP* in FD-derived cells. Increasing the expression level of *IKAP* mRNA over a certain threshold leads to an increase in full-length IKAP protein levels. Although the increase in transcription level was not specific to the *IKAP* gene, its positive effect on the level of IKAP is unquestionable. As PS has been proven safe, it should be tested in FD patients.

The gene expression array analysis revealed that levels of expression of a large number of genes were altered due to PS treatment. It is not surprising that PS affects genes other than *IKAP*, and these effects may be direct or indirect. Kinetin, for example, was shown to affect splicing of other genes [24], and many genes change their expression level in IKAP-deficient cells [20], [21], [39]. Of particular interest in the list of genes demonstrated to be affected is the *TM4SF1* gene, a transmembrane 4L six family member, also known as L6-Ag, that influences cell motility [46], [47]. It is possible that the PS-induced increase in *TM4SF1* mRNA levels increases IKAP levels through regulation involving cell migration, which is impaired in Elongator-depleted cells [20]. Also of interest is the increase in *MYC* expression; this v-myc myelocytomatosis viral oncogene homolog functions as a transcription factor [48]. Since FD is characterized by defects in the Elongator complex, involved in transcription elongation [13], [15], PS may affect IKAP levels through regulation of transcriptional elongation.

Several gene expression profiling involving IKAP were reported. Analysis of cerebrum samples of FD patients revealed down regulation of genes involved in oligodendrocyte differentiation and myelination compared to controls [21]. These genes do not correlate with genes up-regulated after PS treatment. This is probably because PS affects transcription rather than IKAP function directly. It is also possible that these differences are related to the type of cell tested (fibroblasts compared to cerebrum). Also, gene expression analysis of HeLa cells treated with RNAi directed against IKAP revealed that a significant fraction of down-regulated genes encode proteins regulating cell motility, cell proliferation, cellular processes such as autophagy, metabolism and DNA repair; the up-regulated genes were involved in metabolism, transcription and apoptosis [20]. PS presumably does not influence IKAP directly; therefore, it is not unexpected that genes with altered expression as a result of PS treatment are different from genes affected by silencing of IKAP. Microarray analysis performed on induced pluripotent stem cells from FD patients compared to normal controls revealed that down-regulated genes are involved in peripheral neurogenesis and neuronal differentiation [39]. However, the fibroblast cells used in this study were already differentiated, as are most of the cells in the human body. The divergence of our altered genes due to PS treatment from those reported in the literature in cells without IKAP indicate the unique mechanism of action that results in the PS-induced increase in IKAP levels. This body of data also emphasizes the fact that IKAP is a multi-functional protein that contributes to many processes in the cell.

Compounds that increase levels of correctly spliced transcripts have been reported in studies of several disorders that result from exon skipping. Most examples are found in experiments involving models of the neuromuscular disorder spinal muscular atrophy (SMA). Valproic acid [49], sodium butyrate [50], hydroxyurea [51], aclarubicin [52], benzamide M344 [53] and a tetracycline-like compound [54] have all been shown to promote inclusion of exon 7 in the *SMN2* transcript. For FD, several drugs, including kinetin and tocotrienol, increase the levels of wild-type *IKAP* mRNA and protein. In our system, kinetin significantly elevated the level of the normal *IKAP* isoform. It also altered the ratio between the included and the skipped isoforms, shifting the ratio toward inclusion. Kinetin is a plant hormone from the cytokinin family that promotes cell division [55]. It is used in the cosmetic industry as an anti-oxidant with anti-ageing effects [56]. However, kinetin has cytotoxic activity [57], especially at high concentrations [58]. Additionally, in an *in vivo* study treating carriers of FD with kinetin, some adverse effects resulted from treatment [35]. Therefore, kinetin has potential, but its toxicity must be further assessed. Tocotrienols are members of the vitamin E family considered to have neuroprotective and antioxidant properties [59]. The toxicity levels for humans are presently unknown, although estimates have been made based on studies in rats [60]. Tocotrienol, however, did not have an effect in our FD systems (Figure 7B) or in another study [39]. Our data suggest that PS, a non-toxic food supplement already widely used in humans, and its analogs are suitable candidates for testing in FD patients.

## Materials and Methods

### Cell culture and treatment

Control fibroblast cell lines were kindly provided by Prof. Aharon Razin from the Hebrew University Medical School in Jerusalem. FDA and FDB cell lines, as well as the heterozygous cell line, were obtained from the NIGMS Human Genetic Mutant Cell Repository: FDA cell line is the GM00850, FDB is GM02342 and the heterozygous cell line is GM04664 (purchased by Prof. Aharon Razin). The FDD cell line was received from Channa Maayan. All cell lines were immortalized by Ida Vig and Yaniv Lerenthal from Tel Aviv University by transducing the cells with a retroviral vector expressing the catalytic subunit of human telomerase (hTERT), as described previously [61]. HEK 293T cells were obtained from the American Type Culture Collection (CRL-11268). 293T and control fibroblast cell lines were cultured in Dulbecco's modified Eagle's medium (DMEM) with 10% fetal calf serum (FCS), 0.29 mg ml^–1^ L-glutamine, 100 U ml^–1^ penicillin and 0.1 mg ml^–1^ streptomycin at 37°C in a humidified atmosphere with 5% CO_2_. Heterozygous and FD cell lines were cultured in medium containing 20% FCS. Cells were seeded in 6-well or 10-cm plates one day prior to treatment and were ∼80% confluent at the time of treatment.

For the long-term treatment, the cells were seeded and on the next day 100 µg/ml PS was added. Every two days the medium was replaced and fresh PS was added. One week after the initial PS treatment, the cells were split to allow proper growth. The PS treatment was continued for an additional week. mRNA samples were taken 3, 7 and 14 days from the initial treatment.

PS (soy lecithin derived, produced under the name Sharp PS) and Sharp PS GOLD were obtained from Enzymotec (http://www.sharp-ps.com/products/ps/ps.html) as powders. According to the company's recommendations, the powders were dissolved in 95∶5 chloroform/methanol and a stock solution was made at 20 mg/ml. Sharp GPC was obtained from Enzymotec as a viscous liquid and diluted in water. Krill oil was obtained from Enzymotec as a viscous liquid and diluted in corn oil. Kinetin solution was purchased from Sigma. Tocotreinol, full spectrum E with tocotrienols, was produced by Swanson Ultra.

### Semi-quantitative RT-PCR and QPCR analysis of *IKAP* transcripts

After the indicated time, total RNA was extracted from treated and untreated samples using TRI reagent (Sigma). RNA concentrations were determined using a NanoDrop ND-1000 spectrophotometer. For RT-PCR, 2 µg of total RNA were amplified using avian myeloblastosis virus reverse transcriptase (RT-AMV, Roche) with an oligo(dT) reverse primer. Products were amplified with Red Load Taq master mix (Larova) using primers for exon 19 (forward, 5′-CATTACAGGCCGGCCTGAGCAGCA-3′) and exon 21 (reverse, 5′-CTTAGGGTTATGATCATAAATCAGATT-3′) of *IKAP*. The splicing products were separated on 2% agarose gels and were sequenced. Alternative splicing isoforms were quantified using ImageJ.

QPCR was performed using the Stratagene Mx3005P System using the Absolute Blue QPCR SYBR Green ROX mix (Thermo Scientific). Primers used to detect the *IKAP* wild-type isoform (inclusion of exon 20) were exon19F (5′-TTCACGGATTGTCACTGTTGTGCC-3′) and exon20R (5′-TTGTCCAACCACTTCCGAATCTG-3′). For normalization *HPRT* mRNA was amplified using HPRT-F (5′-TGACACTGGCAAAACAATGCA-3′) and HPRT-R (5′-GGTCCTTTTCACCAGCAAGCT-3′) as used in [24]. Detection of the *IKAP* mutant isoform (skipping of exon 20) was performed using primers bridge 19-21F (5′-CACAAAGCTTGTATTACAGACTT-3′) and exon21R (5′-CTTAGGGTTATGATCATAAATCAG-3′). Analysis was performed using the MxPro 4.01 software. All primer pairs yielded a linear standard curve with an R^2^>0.985 and efficiency of reaction between 90–105%. Data was normalized to untreated cells. All experiments were repeated at least three times, and QPCR experiments were performed in triplicate.

### Western blot analysis

Total proteins were extracted from the cells using a hypotonic lysis buffer (50 mM Tris-HCl, pH 7.5, 1% NP40, 150 mM NaCl, 0.1% SDS, 0.5% deoxycholic acid, 1 mM EDTA) containing protease inhibitor and phosphatase inhibitor cocktails I and II (Sigma). After 20 min centrifugation at 14,000 g at 4°C, the supernatant was collected and protein concentrations were measured using BioRad Protein Assay (BioRad). Proteins were separated in an 8% SDS-PAGE and then electroblotted onto a Protran nitrocellulose transfer membrane (Schleicher & Schuell). The membranes were probed with either a goat anti-IKAP antibody (D-17, Santa Cruz) or a mouse anti-IKAP antibody (BD Biosciences) for 12 hr at 4°C, followed by incubation with secondary antibody, donkey anti-goat IgG HRP (Santa Cruz) or goat anti-mouse IgG (Jackson), as appropriate. Immunoblots were visualized by enhanced chemiluminescence (SuperSignal West Pico chemiluminescent substrate; Thermo scientific) and exposure to X-ray film.

### Microarray analysis

Six Human Gene 1.0 microarray chips (Affymetrix) were used: Three chips were used for the analysis of cDNA prepared from FDB cells treated with PS, and three chips were used for the analysis of cDNA prepared from FDB cells treated with the solvent only (used as controls). Cells were treated with 100 µg/ml PS. RNA was extracted 24 hr later using a Qiagen RNeasy Plus mini kit, according to the manufacturer's protocol. cDNA and microarray chips were prepared and hybridized by the Bioinformatics Unit of Tel Aviv University. One chip from each assay was omitted from the analysis because of unsatisfying clustering results.

Analysis of microarray results was done using SAM. The results were validated using QPCR. Primers for microarray QPCR validation are presented in Figure S4. Enrichment analysis of these genes was done using DAVID to cluster differentially up and down-regulated genes into functional categories using GO identification terms, as well as enrichment in signaling pathways using the KEGG pathways database.

### Flow cytometry

Cells were trypsinized, collected in PBS and centrifuged for 10 min at 282 g at 4°C. The cells were then fixed by dropwise addition of the cell suspension into an ice-cold 70% ethanol in PBS with gentle vortexing, and kept overnight at −20°C. Next, the cells were washed with PBS, left for 30 min at 4°C and then suspended in PBS containing 5 µg/ml DNase-free RNase and stained with PI. Sorting was carried out using FACSort flow cytometry (Becton Dickinson) at 10,000 events per sample. Cell cycle analysis was performed with the ModFit software.

## Supporting Information

Figure S1 **PS effect on IKAP mRNA levels in FDD cell line.** FDD cells were treated with 0, 5, 10, 100, 200 and 300 µg/ml PS. RNA was extracted after 24 hr (**A**) and 48 hr (**B**). Left side: QPCR analysis of the level of exon 20 inclusion isoform (wt). Data were normalized to levels in untreated control cells. Right side: RT‐PCR analysis of the splicing of the endogenous *IKAP* mRNA in FD cells. All splicing products were separated on a 2% agarose gel after RT‐PCR reaction using primers to exons 19 and 21. The PCR products were eluted and sequenced. All experiments were repeated independently three times, and the results shown are representative of an average experiment. QPCR experiments were amplified in triplicate; results shown are mean values ± SD.
(TIF)

Figure S2 **Genes up‐ and down‐regulated after PS treatment in FD cells.** Gene expression microarray analysis was performed on cDNA samples from FDB cells treated with PS; data were compared to that from FDB cells treated with the solvent only. All 441 up‐regulated genes and 436 down‐regulated genes identified by significant analysis of microarray (SAM) are listed.
(DOC)

Figure S3 **Complete Gene Ontology enrichment for up‐regulated genes.** Continued from Table 1. GO analysis for up‐regulated genes revealed by microarray analysis following PS treatment of FDB cells. Enriched categories were identified using DAVID to cluster differentially genes into functional categories using GO identification terms. Significant GO enrichment (p‐value <0.05 after FDR multiple testing correction) was observed only for the up‐regulated genes.
(DOC)

Figure S4 **Primers used for validation of microarray results.** Primers forward and reverse used for validation by QPCR of 11 genes from the microarray analysis.
(DOC)

## References

[1] Axelrod FB (2004). Familial dysautonomia.. Muscle Nerve.

[2] Fares F, Badarneh K, Abosaleh M, Harari-Shaham A, Diukman R (2008). Carrier frequency of autosomal-recessive disorders in the Ashkenazi Jewish population: should the rationale for mutation choice for screening be reevaluated?. Prenat Diagn.

[3] Lehavi O, Aizenstein O, Bercovich D, Pavzner D, Shomrat R (2003). Screening for familial dysautonomia in Israel: evidence for higher carrier rate among Polish Ashkenazi Jews.. Genet Test.

[4] Slaugenhaupt SA, Gusella JF (2002). Familial dysautonomia.. Curr Opin Genet Dev.

[5] Axelrod FB, Goldberg JD, Ye XY, Maayan C (2002). Survival in familial dysautonomia: Impact of early intervention.. J Pediatr.

[6] Axelrod FB (2006). A world without pain or tears.. Clin Auton Res.

[7] Anderson SL, Coli R, Daly IW, Kichula EA, Rork MJ (2001). Familial dysautonomia is caused by mutations of the IKAP gene.. Am J Hum Genet.

[8] Rubin BY, Anderson SL (2008). The molecular basis of familial dysautonomia: overview, new discoveries and implications for directed therapies.. Neuromolecular Med.

[9] Cuajungco MP, Leyne M, Mull J, Gill SP, Lu W (2003). Tissue-specific reduction in splicing efficiency of IKBKAP due to the major mutation associated with familial dysautonomia.. Am J Hum Genet.

[10] Slaugenhaupt SA, Blumenfeld A, Gill SP, Leyne M, Mull J (2001). Tissue-specific expression of a splicing mutation in the IKBKAP gene causes familial dysautonomia.. Am J Hum Genet.

[11] Nicholson P, Yepiskoposyan H, Metze S, Zamudio Orozco R, Kleinschmidt N (2010). Nonsense-mediated mRNA decay in human cells: mechanistic insights, functions beyond quality control and the double-life of NMD factors.. Cell Mol Life Sci.

[12] Slaugenhaupt SA, Mull J, Leyne M, Cuajungco MP, Gill SP (2004). Rescue of a human mRNA splicing defect by the plant cytokinin kinetin.. Hum Mol Genet.

[13] Hawkes NA, Otero G, Winkler GS, Marshall N, Dahmus ME (2002). Purification and characterization of the human elongator complex.. J Biol Chem.

[14] Holmberg C, Katz S, Lerdrup M, Herdegen T, Jaattela M (2002). A novel specific role for I kappa B kinase complex-associated protein in cytosolic stress signaling.. J Biol Chem.

[15] Svejstrup JQ (2007). Elongator complex: how many roles does it play?. Curr Opin Cell Biol.

[16] Nguyen L, Humbert S, Saudou F, Chariot A Elongator - an emerging role in neurological disorders.. Trends Mol Med.

[17] Esberg A, Huang B, Johansson MJ, Bystrom AS (2006). Elevated levels of two tRNA species bypass the requirement for elongator complex in transcription and exocytosis.. Mol Cell.

[18] Rahl PB, Chen CZ, Collins RN (2005). Elp1p, the yeast homolog of the FD disease syndrome protein, negatively regulates exocytosis independently of transcriptional elongation.. Mol Cell.

[19] Johansen LD, Naumanen T, Knudsen A, Westerlund N, Gromova I (2008). IKAP localizes to membrane ruffles with filamin A and regulates actin cytoskeleton organization and cell migration.. J Cell Sci.

[20] Close P, Hawkes N, Cornez I, Creppe C, Lambert CA (2006). Transcription impairment and cell migration defects in elongator-depleted cells: implication for familial dysautonomia.. Mol Cell.

[21] Cheishvili D, Maayan C, Smith Y, Ast G, Razin A (2007). IKAP/hELP1 deficiency in the cerebrum of familial dysautonomia patients results in down regulation of genes involved in oligodendrocyte differentiation and in myelination.. Hum Mol Genet.

[22] Cornez I, Creppe C, Gillard M, Hennuy B, Chapelle JP (2008). Deregulated expression of pro-survival and pro-apoptotic p53-dependent genes upon Elongator deficiency in colon cancer cells.. Biochem Pharmacol.

[23] Chen YT, Hims MM, Shetty RS, Mull J, Liu L (2009). Loss of mouse Ikbkap, a subunit of elongator, leads to transcriptional deficits and embryonic lethality that can be rescued by human IKBKAP.. Mol Cell Biol.

[24] Hims MM, Ibrahim EC, Leyne M, Mull J, Liu L (2007). Therapeutic potential and mechanism of kinetin as a treatment for the human splicing disease familial dysautonomia.. J Mol Med.

[25] Vance JE, Steenbergen R (2005). Metabolism and functions of phosphatidylserine.. Prog Lipid Res.

[26] Pepeu G, Pepeu IM, Amaducci L (1996). A review of phosphatidylserine pharmacological and clinical effects. Is phosphatidylserine a drug for the ageing brain?. Pharmacol Res.

[27] U.S. Food and Drug administration website.. http://www.fda.gov/Food/LabelingNutrition/LabelClaims/QualifiedHealthClaims/ucm072999.htm.

[28] Crook TH, Tinklenberg J, Yesavage J, Petrie W, Nunzi MG (1991). Effects of phosphatidylserine in age-associated memory impairment.. Neurology.

[29] Huang da W, Sherman BT, Lempicki RA (2009). Systematic and integrative analysis of large gene lists using DAVID bioinformatics resources.. Nat Protoc.

[30] Crook T, Klatz RM, Goldman R (1998). Treatment of age-related cognitive decline: effects of phosphatidylserine in anti-aging medical therapeutics..

[31] De Jesus Moreno Moreno M (2003). Cognitive improvement in mild to moderate Alzheimer's dementia after treatment with the acetylcholine precursor choline alfoscerate: a multicenter, double-blind, randomized, placebo-controlled trial.. Clin Ther.

[32] Bunea R, El Farrah K, Deutsch L (2004). Evaluation of the effects of Neptune Krill Oil on the clinical course of hyperlipidemia.. Altern Med Rev.

[33] Sampalis F, Bunea R, Pelland MF, Kowalski O, Duguet N (2003). Evaluation of the effects of Neptune Krill Oil on the management of premenstrual syndrome and dysmenorrhea.. Altern Med Rev.

[34] Hims MM, Shetty RS, Pickel J, Mull J, Leyne M (2007). A humanized IKBKAP transgenic mouse models a tissue-specific human splicing defect.. Genomics.

[35] Gold-von Simson G, Goldberg JD, Rolnitzky LM, Mull J, Leyne M (2009). Kinetin in familial dysautonomia carriers: implications for a new therapeutic strategy targeting mRNA splicing.. Pediatr Res.

[36] Anderson SL, Qiu J, Rubin BY (2003). Tocotrienols induce IKBKAP expression: a possible therapy for familial dysautonomia.. Biochem Biophys Res Commun.

[37] Anderson SL, Rubin BY (2005). Tocotrienols reverse IKAP and monoamine oxidase deficiencies in familial dysautonomia.. Biochem Biophys Res Commun.

[38] Anderson SL, Qiu J, Rubin BY (2003). EGCG corrects aberrant splicing of IKAP mRNA in cells from patients with familial dysautonomia.. Biochem Biophys Res Commun.

[39] Lee G, Papapetrou EP, Kim H, Chambers SM, Tomishima MJ (2009). Modelling pathogenesis and treatment of familial dysautonomia using patient-specific iPSCs.. Nature.

[40] Vaisman N, Kaysar N, Zaruk-Adasha Y, Pelled D, Brichon G (2008). Correlation between changes in blood fatty acid composition and visual sustained attention performance in children with inattention: effect of dietary n-3 fatty acids containing phospholipids.. Am J Clin Nutr.

[41] Schreiber S, Kampf-Sherf O, Gorfine M, Kelly D, Oppenheim Y (2000). An open trial of plant-source derived phosphatydilserine for treatment of age-related cognitive decline.. Isr J Psychiatry Relat Sci.

[42] Kidd PM (1996). Phosphatidylserine; Membrane nutrient for Memory. A clinical and mechanistic assesment.. Altern Med Rev.

[43] Ohkubo T, Tanaka Y (2010). Administration of DHA-PS to aged mice was suitable for increasing hippocampal PS and DHA ratio.. J Oleo Sci.

[44] Kingsley M (2006). Effects of phosphatidylserine supplementation on exercising humans.. Sports Med.

[45] Jorissen BL, Brouns F, Van Boxtel MP, Riedel WJ (2002). Safety of soy-derived phosphatidylserine in elderly people.. Nutr Neurosci.

[46] Lekishvili T, Fromm E, Mujoomdar M, Berditchevski F (2008). The tumour-associated antigen L6 (L6-Ag) is recruited to the tetraspanin-enriched microdomains: implication for tumour cell motility.. J Cell Sci.

[47] Storim J, Friedl P, Schaefer BM, Bechtel M, Wallich R (2001). Molecular and functional characterization of the four-transmembrane molecule l6 in epidermal keratinocytes.. Exp Cell Res.

[48] Amin C, Wagner AJ, Hay N (1993). Sequence-specific transcriptional activation by Myc and repression by Max.. Mol Cell Biol.

[49] Brichta L, Hofmann Y, Hahnen E, Siebzehnrubl FA, Raschke H (2003). Valproic acid increases the SMN2 protein level: a well-known drug as a potential therapy for spinal muscular atrophy.. Hum Mol Genet.

[50] Chang JG, Hsieh-Li HM, Jong YJ, Wang NM, Tsai CH (2001). Treatment of spinal muscular atrophy by sodium butyrate.. Proc Natl Acad Sci U S A.

[51] Grzeschik SM, Ganta M, Prior TW, Heavlin WD, Wang CH (2005). Hydroxyurea enhances SMN2 gene expression in spinal muscular atrophy cells.. Ann Neurol.

[52] Andreassi C, Jarecki J, Zhou J, Coovert DD, Monani UR (2001). Aclarubicin treatment restores SMN levels to cells derived from type I spinal muscular atrophy patients.. Hum Mol Genet.

[53] Riessland M, Brichta L, Hahnen E, Wirth B (2006). The benzamide M344, a novel histone deacetylase inhibitor, significantly increases SMN2 RNA/protein levels in spinal muscular atrophy cells.. Hum Genet.

[54] Hastings ML, Berniac J, Liu YH, Abato P, Jodelka FM (2009). Tetracyclines that promote SMN2 exon 7 splicing as therapeutics for spinal muscular atrophy.. Sci Transl Med.

[55] Barciszewski J, Massino F, Clark BF (2007). Kinetin–a multiactive molecule.. Int J Biol Macromol.

[56] Rattan S (2002). N6-Furfuryladenine (Kinetin) as a potential Anti-Aging Molecule.. Journal of Anti-Aging Medicine.

[57] Guillard J, Decrop M, Gallay N, Espanel C, Boissier E (2007). Synthesis and biological evaluation of 7-azaindole derivatives, synthetic cytokinin analogues.. Bioorg Med Chem Lett.

[58] Sharma SP, Kaur P, Rattan SI (1995). Plant growth hormone kinetin delays ageing, prolongs the lifespan and slows down development of the fruitfly Zaprionus paravittiger.. Biochem Biophys Res Commun.

[59] Sen CK, Khanna S, Roy S (2007). Tocotrienols in health and disease: the other half of the natural vitamin E family.. Mol Aspects Med.

[60] Nakamura H, Furukawa F, Nishikawa A, Miyauchi M, Son HY (2001). Oral toxicity of a tocotrienol preparation in rats.. Food Chem Toxicol.

[61] Wood LD, Halvorsen TL, Dhar S, Baur JA, Pandita RK (2001). Characterization of ataxia telangiectasia fibroblasts with extended life-span through telomerase expression.. Oncogene.

