# Supplementary figures and images for: Phosphatidylserine Increases IKBKAP Levels in Familial Dysautonomia Cells
Source: PLoS One. 2010 Dec 29;5(12):e15884. doi: 10.1371/journal.pone.0015884 (PMC3012102; doi:10.1371/journal.pone.0015884)

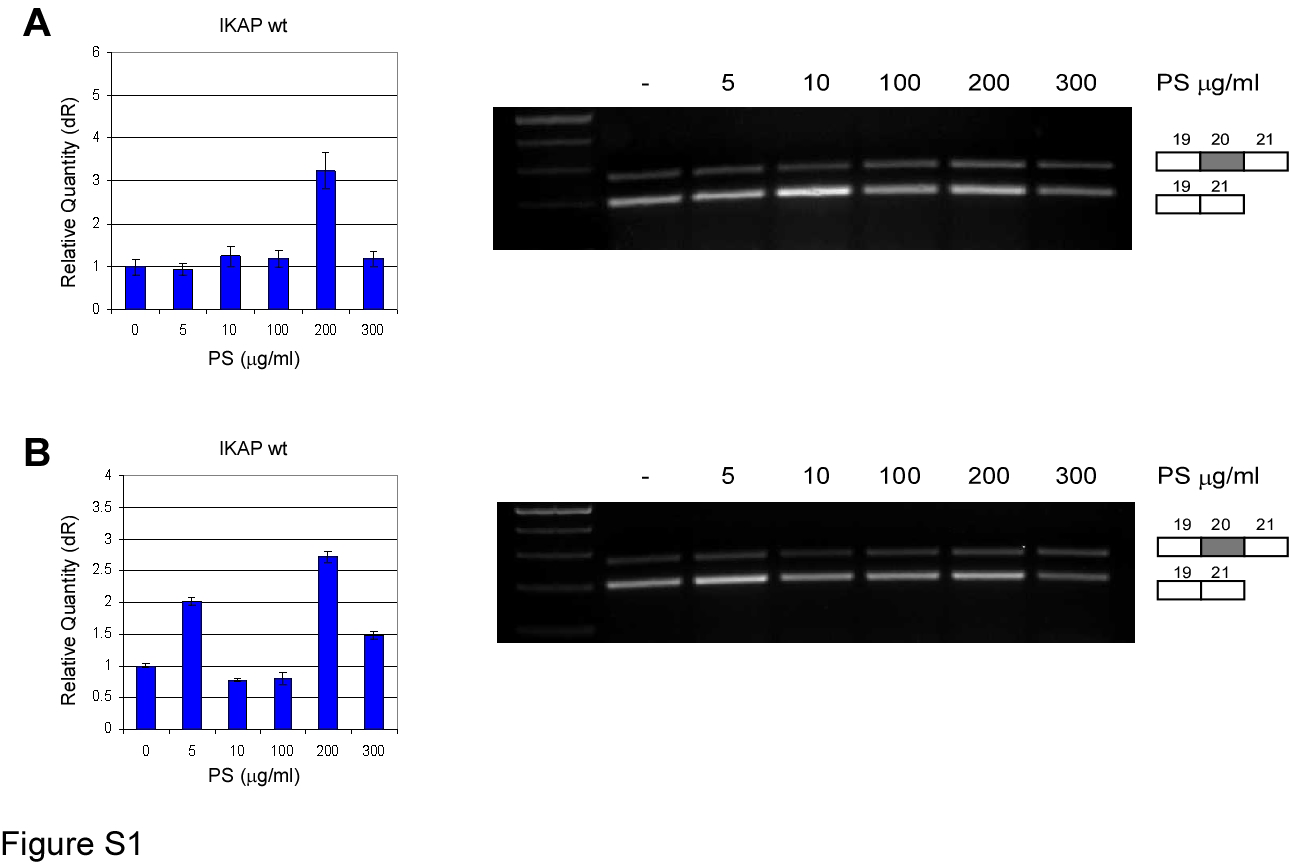

Supplement: Figure S1 — PS effect on IKAP mRNA levels in FDD cell line. FDD cells were treated with 0, 5, 10, 100, 200 and 300 µg/ml PS. RNA was extracted after 24 hr (A) and 48 hr (B). Left side: QPCR analysis of the level of exon 20 inclusion isoform (wt). Data were normalized to levels in untreated control cells. Right side: RT‐PCR analysis of the splicing of the endogenous IKAP mRNA in FD cells. All splicing products were separated on a 2% agarose gel after RT‐PCR reaction using primers to exons 19 and 21. The PCR products were eluted and sequenced. All experiments were repeated independently three times, and the results shown are representative of an average experiment. QPCR experiments were amplified in triplicate; results shown are mean values ± SD. (TIF) [file pone.0015884.s001.tif]
