# Supplementary material for: Phosphatidylserine Increases IKBKAP Levels in Familial Dysautonomia Cells
Source: PLoS One. 2010 Dec 29;5(12):e15884. doi: 10.1371/journal.pone.0015884 (PMC3012102; doi:10.1371/journal.pone.0015884)
Supplement: Figure S2 — Genes up‐ and down‐regulated after PS treatment in FD cells. Gene expression microarray analysis was performed on cDNA samples from FDB cells treated with PS; data were compared to that from FDB cells treated with the solvent only. All 441 up‐regulated genes and 436 down‐regulated genes identified by significant analysis of microarray (SAM) are listed. (DOC) [file pone.0015884.s002.doc]

**Down-**regulated genes

| **Symbol** | **Fold Change** |
| --- | --- |
| RCAN2 | 0.240782 |
| ROBO2 | 0.252412 |
| ITGB8 | 0.255359 |
| CYP7B1 | 0.267464 |
| GALNTL2 | 0.2804 |
| ZNF608 | 0.295399 |
| C6orf138 | 0.308611 |
| APCDD1 | 0.311625 |
| ADH1B | 0.312691 |
| LPAR6 | 0.319334 |
| CCL2 | 0.32526 |
| IL17RB | 0.330035 |
| TCP11L2 | 0.352873 |
| VIT | 0.365822 |
| C7orf69 | 0.367254 |
| THRB | 0.373939 |
| VWA5A | 0.374195 |
| TSHZ2 | 0.386023 |
| PLD1 | 0.390297 |
| PTGFR | 0.406903 |
| SLC9A9 | 0.40831 |
| TMEM119 | 0.409613 |
| MXRA5 | 0.413284 |
| METTL7A | 0.418652 |
| CDON | 0.421091 |
| SIPA1L2 | 0.421521 |
| SEPP1 | 0.422806 |
| C5orf4 | 0.42333 |
| FIBIN | 0.424633 |
| SLCO2A1 | 0.426138 |
| OLFML1 | 0.429432 |
| ADAMTS5 | 0.430949 |
| PAPPA | 0.431251 |
| SPATA18 | 0.435451 |
| FAM19A2 | 0.439047 |
| REV3L | 0.441124 |
| JAM2 | 0.44415 |
| BDKRB2 | 0.449113 |
| TNFRSF19 | 0.450756 |
| CPM | 0.453238 |
| ROBO1 | 0.453688 |
| GNA14 | 0.457408 |
| LOC440104 | 0.459443 |
| DIRC1 | 0.462758 |
| STC2 | 0.464069 |
| CLIC2 | 0.466234 |
| LIPC | 0.471033 |
| ZNF521 | 0.472921 |
| HECW2 | 0.477298 |
| MAP2K6 | 0.477699 |
| STARD5 | 0.47779 |
| GAS1 | 0.479995 |
| OSR2 | 0.480192 |
| CNTN3 | 0.481082 |
| LRRN4CL | 0.481448 |
| MKX | 0.485225 |
| MAP7 | 0.485714 |
| ITIH5 | 0.486434 |
| C5orf41 | 0.487485 |
| SECTM1 | 0.487486 |
| SFRP2 | 0.48891 |
| SLC5A3 | 0.492306 |
| TLE4 | 0.493351 |
| WNT5A | 0.497793 |
| CADM1 | 0.499659 |
| KCNJ2 | 0.502709 |
| CRISPLD2 | 0.505937 |
| ANGPTL2 | 0.50631 |
| ARRDC3 | 0.508552 |
| C1RL | 0.509463 |
| RHOU | 0.510146 |
| PLCD4 | 0.510898 |
| TMEM140 | 0.511061 |
| MID1 | 0.511759 |
| LOC652811 | 0.51275 |
| CXCL6 | 0.51309 |
| PDE4DIP | 0.514306 |
| FAT4 | 0.516491 |
| PLXNC1 | 0.516711 |
| MXD4 | 0.517031 |
| YPEL2 | 0.517832 |
| ARMC9 | 0.517904 |
| KLHL24 | 0.518703 |
| GALNT5 | 0.519138 |
| TNS3 | 0.519354 |
| NBEA | 0.520499 |
| TP53I11 | 0.522914 |
| BHLHE22 | 0.526837 |
| C21orf34 | 0.527278 |
| THBS2 | 0.528813 |
| ATP8B4 | 0.529516 |
| CSF1 | 0.530883 |
| PCDH18 | 0.531758 |
| TMEM26 | 0.532228 |
| CFB | 0.535451 |
| C10orf107 | 0.535707 |
| SERPINF1 | 0.536198 |
| C13orf31 | 0.537143 |
| COL8A1 | 0.541253 |
| ODZ2 | 0.542187 |
| OLFML2B | 0.542491 |
| ADH1A | 0.542799 |
| RCBTB2 | 0.545064 |
| PSD3 | 0.547207 |
| SCN2A | 0.547986 |
| OR2A20P | 0.549723 |
| INSIG1 | 0.550925 |
| GREM2 | 0.553528 |
| AKR1C3 | 0.553998 |
| GRIN2A | 0.55495 |
| BICC1 | 0.556499 |
| ADAM28 | 0.557894 |
| GRIA3 | 0.56167 |
| XG | 0.56365 |
| DMPK | 0.563662 |
| CRABP2 | 0.566533 |
| GABRE | 0.568735 |
| MAFB | 0.568871 |
| OR2A9P | 0.569135 |
| SIX1 | 0.5701 |
| ZMAT3 | 0.574025 |
| RUNX1T1 | 0.574233 |
| LUM | 0.576078 |
| NFATC2 | 0.576467 |
| PDZRN3 | 0.578313 |
| PTGFRN | 0.579678 |
| PTBP2 | 0.580293 |
| LAMB3 | 0.583312 |
| RGL1 | 0.584575 |
| FAM46A | 0.584793 |
| GLT8D2 | 0.586977 |
| PDK2 | 0.589836 |
| CA5B | 0.589959 |
| HSD17B14 | 0.590845 |
| SEMA3D | 0.59131 |
| VGLL3 | 0.592416 |
| PNRC1 | 0.593372 |
| RAPGEF2 | 0.597278 |
| SELENBP1 | 0.599117 |
| ARID5B | 0.602199 |
| PAMR1 | 0.602614 |
| PPAP2B | 0.60296 |
| HBP1 | 0.604865 |
| NDRG1 | 0.606458 |
| LRRN3 | 0.607416 |
| CREB3L1 | 0.609108 |
| S100A3 | 0.611073 |
| ANTXR1 | 0.611263 |
| ORAI3 | 0.611682 |
| SSBP2 | 0.611909 |
| CALCOCO1 | 0.612276 |
| WBP1 | 0.613357 |
| HOXA11 | 0.613763 |
| PAPPAS | 0.616186 |
| TRAF3IP2 | 0.61756 |
| FAM115A | 0.617651 |
| GNAO1 | 0.617821 |
| PBX1 | 0.618096 |
| RNASEL | 0.618099 |
| CCNG1 | 0.618279 |
| NAP1L3 | 0.619128 |
| GTF2IRD2 | 0.619753 |
| FZD2 | 0.620358 |
| DNALI1 | 0.622207 |
| GFRA1 | 0.622487 |
| NEO1 | 0.622775 |
| TGFBR3 | 0.623001 |
| RP11-345P4.4 | 0.624251 |
| FAM110B | 0.62462 |
| CCL7 | 0.625115 |
| MYOCD | 0.625209 |
| CBLN3 | 0.62539 |
| PXK | 0.626943 |
| CLIP3 | 0.62905 |
| OLFML3 | 0.629241 |
| CTSO | 0.629283 |
| PTPLAD2 | 0.63171 |
| LRIG3 | 0.631763 |
| KIT | 0.632001 |
| ALPK2 | 0.632486 |
| SERPING1 | 0.632603 |
| SOCS2 | 0.634588 |
| ZC3H12B | 0.63534 |
| C2orf67 | 0.63539 |
| CYB5D2 | 0.635614 |
| TBC1D17 | 0.638676 |
| PDPN | 0.640205 |
| PDE7B | 0.641272 |
| ZKSCAN1 | 0.641734 |
| FOXO4 | 0.644141 |
| C9orf3 | 0.644954 |
| FUCA1 | 0.64707 |
| RASSF2 | 0.647646 |
| CRABP1 | 0.648168 |
| ACAD11 | 0.648464 |
| HEPH | 0.650833 |
| RPS27L | 0.652662 |
| SYT11 | 0.652847 |
| TBCK | 0.653351 |
| CES2 | 0.653553 |
| ASS1 | 0.654074 |
| PFTK2 | 0.654171 |
| KIAA1377 | 0.654892 |
| 40246 | 0.655647 |
| ENPP5 | 0.656197 |
| DBC1 | 0.657529 |
| HSBP1 | 0.657725 |
| EMX2 | 0.658562 |
| CCNG2 | 0.659096 |
| NFATC4 | 0.659487 |
| TSPAN9 | 0.660241 |
| ACSS3 | 0.6619 |
| LDOC1 | 0.662033 |
| CPT1C | 0.66224 |
| LZTFL1 | 0.662939 |
| VDR | 0.663527 |
| LPAR1 | 0.664077 |
| ZNF436 | 0.665296 |
| PPAP2A | 0.666854 |
| C21orf88 | 0.667454 |
| C16orf45 | 0.668032 |
| LDB1 | 0.669488 |
| NINJ1 | 0.66979 |
| FBLN2 | 0.670361 |
| C7orf63 | 0.672542 |
| TTC39C | 0.673731 |
| LRRC27 | 0.675332 |
| C14orf159 | 0.678105 |
| TENC1 | 0.679413 |
| NBR1 | 0.680141 |
| WBP2 | 0.680543 |
| BSCL2 | 0.681313 |
| COL3A1 | 0.681314 |
| ING4 | 0.683587 |
|  | 0.683897 |
| CD46 | 0.685691 |
| FNDC3B | 0.685726 |
| HOXA9 | 0.687387 |
| DNAJB2 | 0.687405 |
| WIPI1 | 0.68923 |
| PLXNB2 | 0.691066 |
| CLCN6 | 0.691643 |
| PEX11A | 0.69168 |
| CDKN1A | 0.692141 |
| GUCY1A2 | 0.692677 |
| BCAS3 | 0.692885 |
| C11orf49 | 0.693827 |
| PDGFC | 0.693974 |
| MOCOS | 0.69461 |
| KCNK2 | 0.695119 |
| APOBEC3C | 0.695233 |
| DHX40 | 0.695726 |
| CCL8 | 0.696774 |
| KBTBD3 | 0.696963 |
| HMGCL | 0.698128 |
| FAM149B1 | 0.698712 |
| IQCK | 0.703997 |
| AGBL5 | 0.70425 |
| PTPN14 | 0.70537 |
| MR1 | 0.706772 |
| HDAC5 | 0.706834 |
| SESN1 | 0.707191 |
| DKK3 | 0.707374 |
| FAM20A | 0.707645 |
| PIGV | 0.708108 |
| TSPAN31 | 0.708238 |
| TWIST1 | 0.709946 |
| CDC14B | 0.710326 |
| PRICKLE2 | 0.710996 |
| HIST2H2BE | 0.711026 |
| CD109 | 0.71211 |
| MAGED4 | 0.712199 |
| C16orf58 | 0.712694 |
| TMEM176A | 0.714398 |
| CEBPB | 0.715542 |
| AGXT2L2 | 0.715766 |
| TPTE2 | 0.717064 |
| TMEM45A | 0.717281 |
| NBL1 | 0.717286 |
| C22orf9 | 0.717608 |
| TMEM179B | 0.717766 |
| NIPSNAP1 | 0.718192 |
| SVIL | 0.718472 |
| FAM63A | 0.719959 |
| TLE1 | 0.721133 |
| POLI | 0.721154 |
| LOC100131860 | 0.721201 |
| C8orf85 | 0.722199 |
| EDEM2 | 0.722439 |
| ST8SIA1 | 0.723703 |
| TRIM2 | 0.723961 |
| CTDSP2 | 0.725238 |
| SBF2 | 0.72572 |
| ALDH3B1 | 0.727536 |
| GBA | 0.727718 |
| MAPRE2 | 0.727737 |
| LOC541473 | 0.728976 |
| DAZAP2 | 0.729088 |
| CLUAP1 | 0.730636 |
| TMEM106B | 0.730714 |
| OSBPL2 | 0.731297 |
| TBC1D8B | 0.732324 |
| HOXA10 | 0.732365 |
| CAT | 0.733439 |
| SELL | 0.733874 |
| MAP9 | 0.734728 |
| BBS9 | 0.73484 |
| ZNF470 | 0.73515 |
| ZNF189 | 0.735234 |
| ACOX2 | 0.736264 |
| PLK1S1 | 0.738978 |
| IL10RB | 0.739121 |
| PRAF2 | 0.739312 |
| IL1R1 | 0.739413 |
| LOC645212 | 0.741106 |
| RECK | 0.743267 |
| ZMYM2 | 0.743496 |
| PPP1R3B | 0.743808 |
| ATF6B | 0.744156 |
| VASN | 0.744471 |
| TRIM13 | 0.744569 |
| WDR60 | 0.744725 |
| FBXL20 | 0.745511 |
| ANKRA2 | 0.745966 |
| BBS1 | 0.748681 |
| YIPF3 | 0.748873 |
| GPX4 | 0.749277 |
| KCNK1 | 0.749522 |
| MXI1 | 0.750663 |
| SLC44A1 | 0.750768 |
| A4GALT | 0.75178 |
| BAX | 0.752646 |
| TP53INP2 | 0.753983 |
| TRADD | 0.75484 |
| FBLN5 | 0.755025 |
| UNC50 | 0.756201 |
| FAM179B | 0.75659 |
| CA11 | 0.756772 |
| BRE | 0.757722 |
| TMEM47 | 0.758637 |
| SH3BGRL | 0.759414 |
| MGC2752 | 0.761393 |
| NCSTN | 0.761397 |
| CCDC136 | 0.762536 |
| C14orf79 | 0.763414 |
| FUZ | 0.76379 |
| SMPD1 | 0.763979 |
| UBXN1 | 0.764385 |
| TMEM66 | 0.765277 |
| MDM2 | 0.765318 |
| AIG1 | 0.765752 |
| ISCU | 0.766069 |
| KLF9 | 0.766419 |
| ZNF414 | 0.766457 |
| TMEM123 | 0.767818 |
| ATP5I | 0.768313 |
| SH3BP4 | 0.769625 |
| CUBN | 0.770308 |
| TCF4 | 0.770769 |
| GNS | 0.772983 |
| CHN1 | 0.773101 |
| DAB2 | 0.773353 |
| C14orf1 | 0.774631 |
| FKBP9 | 0.774749 |
| CYB5R1 | 0.774898 |
| WDR31 | 0.775101 |
| SDC3 | 0.775601 |
| HCFC1R1 | 0.776996 |
| HEBP2 | 0.778155 |
| FGF10 | 0.778758 |
| TTC28 | 0.779051 |
| ACOT13 | 0.779496 |
| VAT1 | 0.779499 |
| ZNF763 | 0.780992 |
| PCMTD2 | 0.781149 |
| GSTM4 | 0.782784 |
| FCGRT | 0.78337 |
| C1orf85 | 0.783839 |
| SMARCA2 | 0.784759 |
| YPEL5 | 0.784861 |
| SYTL2 | 0.785936 |
| DCTN3 | 0.786011 |
| KCNT2 | 0.787217 |
| CHP | 0.787634 |
| TEF | 0.787666 |
| MAGED2 | 0.789099 |
| MFAP4 | 0.789235 |
| MGC57346 | 0.790861 |
| FAM171B | 0.791141 |
| COQ10A | 0.791658 |
| HEATR6 | 0.791852 |
| SMARCC2 | 0.792596 |
| TMCO3 | 0.794289 |
| ZBTB4 | 0.794369 |
| OLIG2 | 0.794398 |
| PCOLCE | 0.794655 |
| FAM21B | 0.794989 |
| BNIP3L | 0.79542 |
| ATF1 | 0.795897 |
| SLC2A11 | 0.796449 |
| NDST1 | 0.797464 |
| ZNF532 | 0.797866 |
| ITFG1 | 0.799607 |
| EPX | 0.800578 |
| SNX19 | 0.800759 |
| SYS1 | 0.800771 |
| AMZ2 | 0.801333 |
| R3HDM2 | 0.804581 |
| OS9 | 0.804812 |
| FAM21A | 0.805541 |
| JUNB | 0.805558 |
| PCYOX1 | 0.80561 |
| CPT1B | 0.805793 |
| CASD1 | 0.806278 |
| BFSP1 | 0.806932 |
| GHDC | 0.807366 |
| SFXN3 | 0.807734 |
| ARSG | 0.809402 |
| PARP3 | 0.80966 |
| PPP1R3D | 0.814012 |
|  | 0.815011 |
| KLC4 | 0.821895 |
| DNAL4 | 0.823941 |
| HERPUD2 | 0.826163 |
| ATF5 | 0.827724 |
| SDF4 | 0.829235 |
| ARFIP1 | 0.829647 |
| KDELR1 | 0.833328 |
| GPR137 | 0.837064 |
| PSMB6 | 0.838361 |
| SPAG4 | 0.840794 |
| FLJ30430 | 0.842285 |
| RNF214 | 0.843566 |
| CLCNKB | 0.851413 |
| ARNT | 0.859633 |
| SYNPR | 0.861164 |

**Up-regulated genes**

| **Symbol** | **Fold Change** |
| --- | --- |
| UBE2N | 1.274407 |
| ROD1 | 1.274434 |
| ATRIP | 1.278116 |
| KLHL2 | 1.283829 |
| C6orf105 | 1.305919 |
| BUD13 | 1.314561 |
| RNF168 | 1.315579 |
| PTS | 1.317052 |
| TTC4 | 1.319603 |
| IMP4 | 1.321702 |
| TOMM40 | 1.323913 |
| FRMD4A | 1.325566 |
| PGD | 1.329497 |
| PTRH2 | 1.332693 |
| PELI1 | 1.33313 |
| METTL1 | 1.336945 |
| FAM136A | 1.341024 |
| C2orf69 | 1.341772 |
| FOXRED1 | 1.342408 |
| WHAMM | 1.355133 |
| SNHG12 | 1.355996 |
| MNS1 | 1.356978 |
| PSMD14 | 1.360759 |
| TSEN2 | 1.36211 |
| NOM1 | 1.365172 |
| HIST1H2AH | 1.366487 |
| NCAPH2 | 1.367634 |
| SMC2 | 1.369294 |
| POLD1 | 1.369765 |
| ZNF215 | 1.370465 |
| RRP7A | 1.373594 |
| DCAF15 | 1.375096 |
| CHUK | 1.375226 |
| PLXNA2 | 1.378235 |
| SNORD22 | 1.381343 |
| SLC25A30 | 1.381559 |
| SLC25A25 | 1.382119 |
| EIF1AX | 1.382978 |
| NDUFAF4 | 1.386674 |
| WDR43 | 1.388817 |
| CYCS | 1.394304 |
| SNORA61 | 1.394354 |
| MRPS25 | 1.395759 |
| ANAPC1 | 1.398117 |
| MAGI1 | 1.398764 |
| C12orf56 | 1.399051 |
| MYBL1 | 1.400167 |
| E2F3 | 1.400318 |
| MRPL35 | 1.402367 |
| PM20D2 | 1.403699 |
| SNORD76 | 1.406787 |
| PA2G4 | 1.40728 |
| SEH1L | 1.411047 |
| RRP9 | 1.413179 |
| OTUD6B | 1.416268 |
| ATIC | 1.416839 |
| GART | 1.416879 |
| DNAJC9 | 1.417575 |
| CACYBP | 1.41906 |
| YRDC | 1.420254 |
| GEMIN6 | 1.421511 |
| HSPH1 | 1.422168 |
| SNORD50B | 1.423709 |
| PRC1 | 1.424742 |
| SFRS7 | 1.428532 |
| VANGL1 | 1.433179 |
| C1orf128 | 1.436849 |
| TSR1 | 1.436906 |
| UTP18 | 1.437445 |
| ZNF828 | 1.44022 |
| FAM57A | 1.440809 |
| FAM111A | 1.44102 |
| OSGEPL1 | 1.441091 |
| ZBTB9 | 1.442658 |
| THOC4 | 1.443944 |
| ZNF473 | 1.444416 |
| HN1L | 1.445547 |
| DLGAP5 | 1.445963 |
| CCT5 | 1.446385 |
| FAM64A | 1.447864 |
| CEP152 | 1.449955 |
| PRMT3 | 1.451255 |
| ARL13B | 1.45141 |
| CCDC34 | 1.452037 |
| PDCD11 | 1.454036 |
| CCDC77 | 1.458214 |
| SNORD74 | 1.458609 |
| ATAD3B | 1.459291 |
| XPO1 | 1.45989 |
| TRMT6 | 1.460113 |
| DLG1 | 1.460246 |
| FARSB | 1.462212 |
| RAP1GDS1 | 1.462713 |
| STK39 | 1.464558 |
| FAM176A | 1.464767 |
| TFAM | 1.466048 |
| PPA1 | 1.466713 |
| SMC6 | 1.467786 |
| ACD | 1.468166 |
| KATNAL1 | 1.469634 |
| CCT6A | 1.471038 |
| H2AFX | 1.473994 |
| SSR3 | 1.474164 |
| KLF6 | 1.474885 |
| LCP1 | 1.476058 |
| TAF5 | 1.477371 |
| NUPL1 | 1.479734 |
| PTDSS1 | 1.480346 |
| C8orf48 | 1.482331 |
| LOC643015 | 1.483791 |
| CASP8AP2 | 1.483943 |
| NUP88 | 1.484014 |
| ZNF367 | 1.484756 |
| C12orf48 | 1.484973 |
| ZNF738 | 1.48503 |
| GEN1 | 1.486844 |
| CKS1B | 1.490408 |
| E2F8 | 1.491634 |
| C10orf119 | 1.493161 |
| CWF19L1 | 1.497201 |
| TRIM59 | 1.498643 |
| TIMM10 | 1.499501 |
| KIF4A | 1.503599 |
| RIMKLB | 1.504144 |
| RPL22L1 | 1.504516 |
| PALB2 | 1.504885 |
| ARHGAP19 | 1.505155 |
| TOP2A | 1.505293 |
| CBFB | 1.506696 |
| HAT1 | 1.50676 |
| POLR3G | 1.507292 |
| SLFN11 | 1.508246 |
| NOC3L | 1.508799 |
| MYO19 | 1.509955 |
| CSE1L | 1.511576 |
| LIG1 | 1.511899 |
| IFRD2 | 1.513292 |
| CTNNAL1 | 1.514976 |
| ZNF85 | 1.516279 |
| PFAS | 1.520591 |
| DEM1 | 1.523263 |
| DKC1 | 1.523661 |
| PLCXD1 | 1.527552 |
| NARG1 | 1.529592 |
| CCDC75 | 1.531373 |
| PAK1 | 1.534508 |
| TEX10 | 1.5384 |
| GTPBP4 | 1.541933 |
| SCG5 | 1.544597 |
| NUP50 | 1.550095 |
| C10orf78 | 1.550502 |
| ACAT2 | 1.55141 |
| OIP5 | 1.552763 |
| POP1 | 1.555372 |
| MIS12 | 1.556743 |
| XPO5 | 1.558867 |
| MAK16 | 1.559258 |
| TNFAIP3 | 1.56136 |
| ITPRIP | 1.561377 |
| NOP16 | 1.561855 |
| NOLC1 | 1.562494 |
| PAK1IP1 | 1.563586 |
| MPP6 | 1.563751 |
| NOP56 | 1.56608 |
| TNFAIP8L3 | 1.567471 |
| UTP15 | 1.569097 |
| C4orf46 | 1.570238 |
| SH3RF1 | 1.571919 |
| FKBP5 | 1.577961 |
| NOP2 | 1.578957 |
| NOL11 | 1.581745 |
| DEPDC1 | 1.587098 |
| EZR | 1.587338 |
| TRAIP | 1.587427 |
| DNMT1 | 1.588408 |
| EHD4 | 1.590067 |
| MRTO4 | 1.591515 |
| CCDC138 | 1.593863 |
| PIGW | 1.599493 |
| ANLN | 1.600136 |
| CHEK1 | 1.603527 |
| TMC7 | 1.606432 |
| AGPAT5 | 1.608848 |
| C16orf61 | 1.608856 |
| ADAT2 | 1.60971 |
| DUSP5 | 1.610673 |
| FAM40B | 1.613858 |
| EBNA1BP2 | 1.615157 |
| ECT2 | 1.615819 |
| AURKA | 1.622446 |
| SPC24 | 1.624274 |
| KIF18B | 1.624489 |
| LRRC8C | 1.625648 |
| CDCA7L | 1.629861 |
| YWHAH | 1.630522 |
| DCK | 1.633162 |
| POLE | 1.633432 |
| NOP58 | 1.637256 |
| FBXO45 | 1.637763 |
| ABCE1 | 1.637878 |
| UMPS | 1.638597 |
| HIST1H2BN | 1.641003 |
| ANKRD32 | 1.642947 |
| RACGAP1 | 1.648639 |
| POLE3 | 1.653367 |
| CENPE | 1.65458 |
|  | 1.655355 |
| C1orf135 | 1.663484 |
| CDT1 | 1.668578 |
| SUV39H2 | 1.678445 |
| RNF219 | 1.678917 |
| G2E3 | 1.678949 |
| CHAC2 | 1.678995 |
| SAAL1 | 1.680505 |
| DCTPP1 | 1.680554 |
| CCNA2 | 1.683611 |
| NT5DC3 | 1.687644 |
| ORC5L | 1.68857 |
| GEMIN4 | 1.691666 |
| CASC5 | 1.696271 |
| TMEM48 | 1.700348 |
| DONSON | 1.706346 |
| UBASH3B | 1.710213 |
| PARP2 | 1.711409 |
| SLC7A6 | 1.712171 |
| ARHGAP11A | 1.717271 |
| UCHL5 | 1.717274 |
| SSX2IP | 1.719018 |
| GABBR2 | 1.724145 |
| BYSL | 1.7267 |
| HAUS8 | 1.726937 |
| SCML2 | 1.728154 |
| NCAPG2 | 1.729241 |
| SNORD30 | 1.729424 |
| CCDC109B | 1.731257 |
| RMI1 | 1.731627 |
| FANCC | 1.731943 |
| MTAP | 1.732006 |
| MAD2L1 | 1.73859 |
| KIF11 | 1.738986 |
| LRP8 | 1.73992 |
| TXNRD1 | 1.740394 |
| UBR7 | 1.74151 |
| NUP107 | 1.741664 |
| SLC8A1 | 1.742692 |
| MTHFD1 | 1.743964 |
| TPX2 | 1.745679 |
| B3GNT2 | 1.750997 |
| CDCA8 | 1.751455 |
| HIST1H2AI | 1.761748 |
| DDX21 | 1.762027 |
| FGF5 | 1.76766 |
| SPHK1 | 1.769696 |
| C4orf21 | 1.770035 |
| FANCM | 1.770938 |
| EPHB1 | 1.77142 |
| ZWILCH | 1.772382 |
| SOAT1 | 1.772525 |
| GPN3 | 1.773363 |
| CEP78 | 1.776559 |
| SPATA5 | 1.77688 |
| URB2 | 1.780648 |
| C14orf145 | 1.782338 |
| KRTAP2-4 | 1.783442 |
| PPAT | 1.791405 |
| KIF14 | 1.792224 |
| TTK | 1.792491 |
| NETO2 | 1.795541 |
| HIST1H2AL | 1.802126 |
| PBK | 1.804449 |
| HIST1H3H | 1.805029 |
| GINS4 | 1.807686 |
| TIMELESS | 1.809162 |
| TMEM194A | 1.810979 |
| USP1 | 1.811152 |
| TRIP13 | 1.811352 |
| KIF20B | 1.813176 |
| C13orf34 | 1.815518 |
| UAP1 | 1.816167 |
| HIST2H3A | 1.816493 |
| POLR1E | 1.818313 |
| HIST1H4E | 1.824865 |
| CDC7 | 1.832206 |
| GSG2 | 1.833871 |
| SHCBP1 | 1.834114 |
| C1orf112 | 1.834951 |
| TES | 1.835971 |
| PLEK2 | 1.843966 |
| KRTAP2-1 | 1.844746 |
| KIF23 | 1.84543 |
| ESPL1 | 1.854213 |
| PRPF4 | 1.85538 |
| CALB2 | 1.855781 |
|  | 1.856892 |
| NUF2 | 1.866307 |
| GGCT | 1.872793 |
| CDC25A | 1.87602 |
| NDC80 | 1.886193 |
| EZH2 | 1.889135 |
| TMPO | 1.89354 |
| ZWINT | 1.894479 |
| EXOSC2 | 1.897379 |
| KIAA0101 | 1.897718 |
| RFWD3 | 1.90073 |
| E2F7 | 1.902531 |
| MCM7 | 1.915846 |
| WDR4 | 1.919218 |
| CEP55 | 1.921199 |
| SKA3 | 1.921596 |
| CHAF1A | 1.921708 |
| KCNQ5 | 1.925733 |
| FANCG | 1.928711 |
| LOC100129478 | 1.929363 |
| CORO2B | 1.930879 |
| KNTC1 | 1.932143 |
| SNORD26 | 1.934905 |
| NUP155 | 1.936629 |
| RRS1 | 1.943369 |
| FAM72A | 1.947484 |
| KIAA1524 | 1.957051 |
| UHRF1 | 1.958398 |
| MYC | 1.958482 |
| SPC25 | 1.959831 |
| CENPJ | 1.979377 |
| PHF17 | 1.981663 |
| DKK1 | 1.982883 |
| SNORD78 | 1.983105 |
| POLA1 | 1.998286 |
| KIFC1 | 1.998704 |
| FANCI | 2.002693 |
| HIST1H4F | 2.007593 |
| C21orf45 | 2.019466 |
| PVR | 2.021947 |
| SKA1 | 2.024372 |
| CTPS | 2.0291 |
| SGOL1 | 2.032696 |
| EME1 | 2.032946 |
| CDCA4 | 2.035715 |
| CDCA2 | 2.036381 |
| FANCA | 2.038574 |
| BARD1 | 2.042501 |
| PSMC3IP | 2.044602 |
| SNORD44 | 2.051524 |
| MSH2 | 2.051623 |
| HJURP | 2.053779 |
| RAD54B | 2.069105 |
| C13orf27 | 2.07171 |
| MCM2 | 2.071719 |
| RFC2 | 2.076248 |
| SNORD31 | 2.079097 |
| DSCC1 | 2.081567 |
| CDC2 | 2.100351 |
| CDCA5 | 2.103238 |
| ERI1 | 2.104471 |
| DCLRE1B | 2.105496 |
| SLC29A1 | 2.107359 |
| HIST1H4D | 2.10819 |
| MLF1IP | 2.109925 |
| LMNB1 | 2.116568 |
| PRIM1 | 2.130573 |
| MCM3 | 2.140509 |
| NCAPH | 2.14957 |
| C11orf82 | 2.150979 |
| HAUS6 | 2.153521 |
| C5orf34 | 2.168956 |
| TIPIN | 2.171532 |
| BUB1B | 2.183658 |
| CENPK | 2.184044 |
|  | 2.185919 |
| MYBL2 | 2.189541 |
| MCM8 | 2.194575 |
| ODC1 | 2.198704 |
| CENPI | 2.202784 |
| MELK | 2.207591 |
| NP | 2.220349 |
| HIST1H2BH | 2.222013 |
| FEN1 | 2.225337 |
| RFC3 | 2.228229 |
| C3HC4 | 2.234499 |
| MCM6 | 2.239157 |
| ORC1L | 2.241298 |
| C5orf30 | 2.245636 |
| C6orf167 | 2.252447 |
| POLA2 | 2.257385 |
| ATAD2 | 2.25753 |
| GINS3 | 2.277468 |
| C15orf42 | 2.279523 |
| ERCC6L | 2.280474 |
| FBXO5 | 2.282465 |
| DIAPH3 | 2.285759 |
| MGAT5 | 2.2906 |
| PDSS1 | 2.298052 |
| PLK4 | 2.306059 |
| WDR76 | 2.319857 |
| HIST1H4C | 2.35271 |
| TMEM200A | 2.362013 |
| HIST2H3D | 2.362329 |
| VRK1 | 2.371848 |
| CHAF1B | 2.381064 |
| TCF19 | 2.385766 |
| FIGNL1 | 2.394293 |
| THBD | 2.403168 |
| ATAD5 | 2.430114 |
| POLE2 | 2.434753 |
| HIST1H3F | 2.437307 |
| NEIL3 | 2.460197 |
| LIFR | 2.473 |
| RBBP8 | 2.474948 |
| MTBP | 2.483377 |
| ORC6L | 2.523465 |
| RAD18 | 2.528408 |
| LOC162632 | 2.549471 |
| SNORD25 | 2.575859 |
| RAD51 | 2.598382 |
| ANGPTL4 | 2.631914 |
| WDHD1 | 2.634982 |
| XRCC2 | 2.642936 |
| GFPT2 | 2.671157 |
| RAD51AP1 | 2.679401 |
| CDC45L | 2.685236 |
| GINS1 | 2.728706 |
| PDLIM5 | 2.742308 |
| CLSPN | 2.742457 |
| PODXL | 2.754789 |
| EXO1 | 2.76335 |
| HELLS | 2.768321 |
| MCM10 | 2.779174 |
| HIST1H4B | 2.808183 |
| RFC4 | 2.827534 |
| DNA2 | 2.830323 |
| HIST1H3J | 2.837777 |
| AGPAT9 | 2.88638 |
| OPCML | 2.925062 |
| TM4SF1 | 2.958657 |
| INHBA | 2.965755 |
| FAM111B | 2.968153 |
| BLM | 2.972919 |
| FANCB | 3.097497 |
| BRIP1 | 3.195171 |
| SEMA7A | 3.391645 |
| LOC554202 | 3.413137 |
