# Supplementary material for: Phosphatidylserine Increases IKBKAP Levels in Familial Dysautonomia Cells
Source: PLoS One. 2010 Dec 29;5(12):e15884. doi: 10.1371/journal.pone.0015884 (PMC3012102; doi:10.1371/journal.pone.0015884)
Supplement: Figure S3 — Complete Gene Ontology enrichment for up‐regulated genes. Continued from Table 1. GO analysis for up‐regulated genes revealed by microarray analysis following PS treatment of FDB cells. Enriched categories were identified using DAVID to cluster differentially genes into functional categories using GO identification terms. Significant GO enrichment (p‐value <0.05 after FDR multiple testing correction) was observed only for the up‐regulated genes. (DOC) [file pone.0015884.s003.doc]

**Figure S3: GO analysis for up-regulated genes**

| **Cluster 5** | **Enrichment score- 10.06** | | |
| --- | --- | --- | --- |
| **GO term** | **p-value^1^** | **# genes^2^** | |
| ATP binding | 2.25E-09 | 73 | |
| Adenyl ribonucleotide binding | 4.33E-09 | 73 | |
| Adenyl nucleotide binding | 1.86E-08 | 74 | |
| Nucleoside binding | 1.91E-08 | 75 | |
| Purine nucleoside binding | 3.82E-08 | 74 | |
| Ribonucleotide binding | 1.31E-06 | 77 | |
| Purine ribonucleotide binding | 1.31E-06 | 77 | |
| Purine nucleotide binding | 4.02E-06 | 78 | |
| Nucleotide binding | 7.42E-06 | 86 | |
| **Cluster 6** | **Enrichment score- 8.63** | | |
| **GO term** | **p-value^1^** | | **# genes^2^** |
| Regulation of cell cycle | 3.82E-10 | | 35 |
| Cell cycle checkpoint | 3.59E-09 | | 19 |
| Regulation of mitotic cell cycle | 1.31E-04 | | 18 |
| Regulation of cell cycle process | 5.88E-04 | | 15 |
| Mitotic cell cycle checkpoint | 0.00873 | | 9 |
| **Cluster 7** | **Enrichment score- 6.03** | | |
| **GO term** | **p-value^1^** | | **# genes^2^** |
| Spindle | 8.88E-10 | | 23 |
| Microtubule cytoskeleton | 1.03E-05 | | 36 |
| Microtubule-based process | 0.002793 | | 21 |
| Cytoskeletal part | 0.008227 | | 43 |
| Microtubule organizing center | 0.01216 | | 19 |
| Centrosome | 0.036647 | | 17 |
| **Cluster 8** | **Enrichment score- 5.79** | | |
| **GO term** | **p-value^1^** | | **# genes^2^** |
| Spindle organization | 4.44E-07 | | 13 |
| Microtubule-based process | 0.002793 | | 21 |
| Microtubule cytoskeleton organization | 0.012649 | | 15 |
| Mitotic spindle organization | 0.026073 | | 6 |
| **Cluster 9** | **Enrichment score- 5.72** | | |
| **GO term** | **p-value^1^** | | **# genes^2^** |
| Cell cycle checkpoint | 3.59E-09 | | 19 |
| DNA integrity checkpoint | 0.03783 | | 9 |
| **Cluster 10** | **Enrichment score- 5.576** | | |
| **GO term** | **p-value^1^** | | **# genes^2^** |
| DNA recombination | 2.08E-04 | | 15 |
| Meiosis | 6.26E-04 | | 14 |
| M phase of meiotic cell cycle | 6.26E-04 | | 14 |
| Meiotic cell cycle | 7.95E-04 | | 14 |
| **Cluster 11** | **Enrichment score- 5.575** | | |
| **GO term** | **p-value^1^** | | **# genes^2^** |
| Ribosome biogenesis | 7.25E-08 | | 20 |
| Ribonucleoprotein complex biogenesis | 1.03E-05 | | 21 |
| ncRNA processing | 1.06E-04 | | 20 |
| rRNA processing | 2.94E-04 | | 14 |
| rRNA metabolic process | 4.90E-04 | | 14 |
| ncRNA metabolic process | 6.22E-04 | | 21 |
| **Cluster 12** | **Enrichment score- 5.55** | | |
| **GO term** | **p-value^1^** | | **# genes^2^** |
| Macromolecular complex assembly | 1.23E-04 | | 40 |
| Macromolecular complex subunit organization | 2.42E-04 | | 41 |
| Cellular macromolecular complex assembly | 0.002163 | | 24 |
| Cellular macromolecular complex subunit organization | 0.004639 | | 25 |
| **Cluster 13** | **Enrichment score- 5.37** | | |
| **GO term** | **p-value^1^** | | **# genes^2^** |
| Chromosome organization | 7.84E-10 | | 42 |
| DNA packaging | 2.37E-06 | | 18 |
| Protein-DNA complex assembly | 0.001885 | | 13 |
| Cellular macromolecular complex assembly | 0.002163 | | 24 |
| Chromatin | 0.001978 | | 18 |
| Cellular macromolecular complex subunit organization | 0.004639 | | 25 |
| Protein-DNA complex | 0.020181 | | 11 |
| **Cluster 14** | **Enrichment score- 4.87** | | |
| **GO term** | **p-value^1^** | | **# genes^2^** |
| Mitotic sister chromatid segregation | 2.33E-08 | | 13 |
| Sister chromatid segregation | 3.37E-08 | | 13 |
| **Cluster 15** | **Enrichment score- 4.69** | | |
| **GO term** | **p-value^1^** | | **# genes^2^** |
| DNA strand elongation during DNA replication | 0.002265 | | 5 |
| DNA strand elongation | 0.00667 | | 5 |
| **Cluster 16** | **Enrichment score- 4.69** | | |
| **GO term** | **p-value^1^** | | **# genes^2^** |
| Double-strand break repair | 2.35E-05 | | 13 |
| DNA recombination | 2.08E-04 | | 15 |
| Recombinational repair | 0.005019 | | 7 |
| Double-strand break repair via homologous recombination | 0.005019 | | 7 |
| **Cluster 17** | **Enrichment score- 4.19** | | |
| **GO term** | **p-value^1^** | | **# genes^2^** |
| Replication fork | 1.02E-06 | | 11 |
| Protein-DNA complex | 0.020181 | | 11 |
| **Cluster 18** | **Enrichment score- 3.46** | | |
| **GO term** | **p-value^1^** | | **# genes^2^** |
| DNA-dependent ATPase activity | 0.003315 | | 10 |
| **Cluster 19** | **Enrichment score- 3.22** | | |
| **GO term** | **p-value^1^** | | **# genes^2^** |
| Microtubule-based process | 0.002793 | | 21 |
| **Cluster 20** | **Enrichment score- 3.2** | | |
| **GO term** | **p-value^1^** | | **# genes^2^** |
| Nucleotide-excision repair, DNA gap filling | 0.002381 | | 7 |
| **Cluster 21** | **Enrichment score- 2.99** | | |
| **GO term** | **p-value^1^** | | **# genes^2^** |
| DNA replication initiation | 0.001571 | | 7 |
| **Cluster 22** | **Enrichment score- 2.14** | | |
| **GO term** | **p-value^1^** | | **# genes^2^** |
| Pore complex | 0.048588 | | 11 |
| **Cluster 23** | **Enrichment score- 2.06** | | |
| **GO term** | **p-value^1^** | | **# genes^2^** |
| Regulation of cell cycle process | 5.88E-04 | | 15 |

^1^FDR multiple testing correction

^2^number of identified genes in Gene Ontology (GO) category

Enriched categories were identified using DAVID; See supplementary file 3 for complete tables.
