# Supplementary material for: Phosphatidylserine Increases IKBKAP Levels in Familial Dysautonomia Cells
Source: PLoS One. 2010 Dec 29;5(12):e15884. doi: 10.1371/journal.pone.0015884 (PMC3012102; doi:10.1371/journal.pone.0015884)
Supplement: Figure S4 — Primers used for validation of microarray results. Primers forward and reverse used for validation by QPCR of 11 genes from the microarray analysis. (DOC) [file pone.0015884.s004.doc]

**Figure S4: Primers used for validation of microarray results.**

| **Gene** | **Primer F** | **Primer R** |
| --- | --- | --- |
| YWHAH | ATTTCCAGTATGAGAGCAAGG | CTTGTAGGCAGCTTCAGAAG |
| TM4SF1 | ATGCTTTCTTCTGTATTGGCTG | TCCACTGGCCGAGGGAATC |
| MYC | AACGTTAGCTTCACCAACAGG | TCGCTCTGCTGCTGCTGC |
| DCTPP1 | GCCTCCATGCTGAGTTTGCT | AAGAGTTCTGCCAGCTCCC |
| BLM | GTTTAGCATGAGCTTTAACAGAC | ATGGGTGGTGCTTTCTGATC |
| BRIP1 | AGCAGATGAGGGCGTAAGTG | AGTTCCTTGGTTCATGTCATTG |
| RCAN2 | TTGAGGGACTGTTTCGGAC | CTCGGGCTGCAGATTTAGG |
| ROBO2 | TCTTCGCCAGGAGGACTTTC | TCAATGGTGGGCGTTGGC |
| CYP7B1 | GTGAGCCTCCATTGATAAAAG | CACCATGTTGCTTTTGAAGTG |
| ITGB8 | ATTTCATTTCAGGTGGATCAAG | GTATTATAACATGCACAGATGG |
| LZIC | TGATACAGATGAATATGAAGAAACC | TCTACCAAAGTCATATTTCCAGAC |
